# Identification of the Spinal Expression Profile of Non-coding RNAs Involved in Neuropathic Pain Following Spared Nerve Injury by Sequence Analysis

**DOI:** 10.3389/fnmol.2017.00091

**Published:** 2017-04-03

**Authors:** Jun Zhou, Qingming Xiong, Hongtao Chen, Chengxiang Yang, Youling Fan

**Affiliations:** ^1^Department of Anesthesiology, The First People's Hospital of FoshanFoshan, China; ^2^Department of Anesthesiology, Eighth People's Hospital of GuangzhouGuangzhou, China; ^3^Department of Anesthesiology, Panyu Central HospitalGuangzhou, China

**Keywords:** neuropathic pain, non-coding RNAs(ncRNAs), sequencing, spinal cord, expression profiles

## Abstract

Neuropathic pain (NP) is caused by damage to the nervous system, resulting in aberrant pain, which is associated with gene expression changes in the sensory pathway. However, the molecular mechanisms are not fully understood. A non-coding Ribose Nucleic Acid (ncRNA) is an RNA molecule that is not translated into a protein. NcRNAs are involved in many cellular processes, and mutations or imbalances of the repertoire within the body can cause a variety of diseases. Although ncRNAs have recently been shown to play a role in NP pathogenesis, the specific effects of ncRNAs in NP remain largely unknown. In this study, sequencing analysis was performed to investigated the expression patterns of ncRNAs in the spinal cord following spared nerve injury-induced NP. A total of 134 long non-coding RNAs (lncRNAs), 12 microRNAs (miRNAs), 188 circular RNAs (circRNAs) and 1066 mRNAs were significantly regulated at 14 days after spared nerve injury (SNI) surgery. Next, quantitative real-time polymerase chain reaction (PCR) was performed to validate the expression of selected lncRNAs, miRNAs, circRNAs, and mRNAs. Bioinformatics tools and databases were employed to explore the potential ncRNA functions and relationships. Our data showed that the most significantly involved pathways in SNI pathogenesis were ribosome, PI3K-Akt signaling pathway, focal adhesion, ECM-receptor interaction, amoebiasis and protein digestion and absorption. In addition, the lncRNA-miRNA-mRNA and circRNA-miRNA-mRNA network of NP was constructed. This is the first study to comprehensively identify regulated ncRNAs of the spinal cord and to demonstrate the involvement of different ncRNA expression patterns in the spinal cord of NP pathogenesis by sequence analysis. This information will enable further research on the pathogenesis of NP and facilitate the development of novel NP therapeutics targeting ncRNAs.

## Introduction

Neuropathic pain (NP) is one type of chronic pain and is caused by primary damage and dysfunction of the nervous system, which is characterized by dysesthesia, hyperalgesia, and allodynia (Gaskin and Richard, 2012; Finnerup et al., 2016). The number of patients with this type of pain has increased rapidly in recent years (Langley et al., 2010, 2013). Indeed, the mechanisms of NP remain poorly understood, but it is known to involve nerve injury, inflammation ectopic discharge, anatomical remodeling, N Methyl D Aspartate (NMDA) and P2X receptors, etc. (Chen et al., 2012, 2013; Detloff et al., 2014). Thus, an improved knowledge of the pathogenesis of NP is crucial for the development of an effective therapeutic strategy to prevent NP and improve the curative effect. A better understanding of the genetic and various neurobiological bases could provide the clinician with important diagnosis and treatment tools.

Non-coding Ribose Nucleic Acids (ncRNAs) comprise a class of RNA molecules that typically do not encode proteins but functionally regulate protein expression (Mattick and Makunin, 2006). Based on their size, ncRNAs can be subdivided into small ncRNAs (<200 nucleotides long), including microRNAs (miRNAs) and long ncRNAs (lncRNAs), with a length >200 bp, as well as circular RNAs (circRNA) consisting of a closed continuous loop (Thum, 2014; Thum and Condorelli, 2015). It has been speculated that these ncRNAs are emerging key regulators of gene expression under physiological and pathological conditions (Wang and Chang, 2011; Yang et al., 2014; Li et al., 2016). Moreover, emerging data have shown that ncRNAs are of crucial importance in various types of pain, particularly NP (Chen et al., 2014; Shao et al., 2015; Zhang and Banerjee, 2015; Wang et al., 2016). However, the regulatory functions of ncRNAs in NP and their underlying functional mechanisms have not been systematically described. Thus, a comprehensive forecasting and analysis of the ncRNAs underlying the progression of NP are essential for the development of effective strategies to treat this troublesome disorder and to prevent its progression.

The spared nerve injury (SNI) model induces symptoms of NP, such as mechanical allodynia due to tactile stimuli that do not normally provoke a painful response (Costigan et al., 2009). The advantages of the SNI model are the robustness of the response and the lack of a requirement for expert microsurgical skills (Decosterd and Woolf, 2008). In this study, we predicted and analyzed ncRNAs of the spinal cord in SNI-induced NP using RNA sequencing techniques.

## Results

### Model identification of neuropathic pain

**Figure 1:**
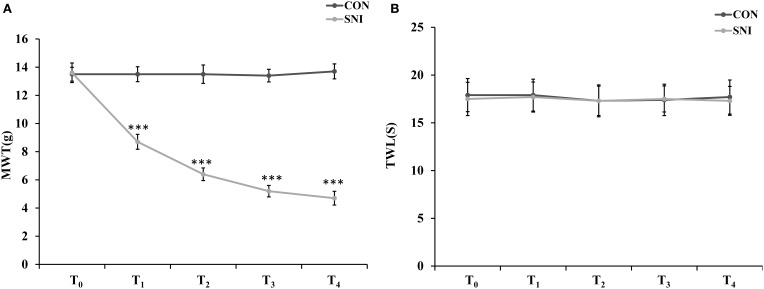
**Nociceptive behavior developed in SNI model rats**. MWT (mechanical withdrawal threshold) in each time points **(A)**, TWL (thermal withdrawal latency) in each time points **(B)**. *n* = 6, ^***^p < 0.001 compared to the CON group.

To determine the effect of NP on ncRNAs in the spinal cord, the SNI model was generated using SD rats. In the SNI model in rats, the common peroneal and tibial nerves are injured, producing consistent and reproducible pain hypersensitivity in the territory of the spared sural nerve (Bourquin et al., 2006). Twelve rats were randomly divided into the CON group (*n* = 6) and SNI group (*n* = 6). The rats were evaluated by mechanical allodynia and thermal sensitivity using von Frey and Hargreaves plantar tests at 3 h before and 1, 3, 7, and 14 days after surgery (T_0_, T_1_, T_2_, T_3_, and T_4_). Three rats were randomly sacrificed and samples of L_4−5_ spinal cord were collected after the detection of pain threshold at T_4_ in each group. All SNI model rats developed mechanical allodynia in the ipsilateral side at 1, 3, 7, and 14 days after SNI surgery compared to the CON group (Figure 1A). The SNI model did not modify the thermal sensitivity of the rats at any time point (Figure 1B).

### Differentially expressed (DE) ncRNAs and mRNAs

**Figure 2:**
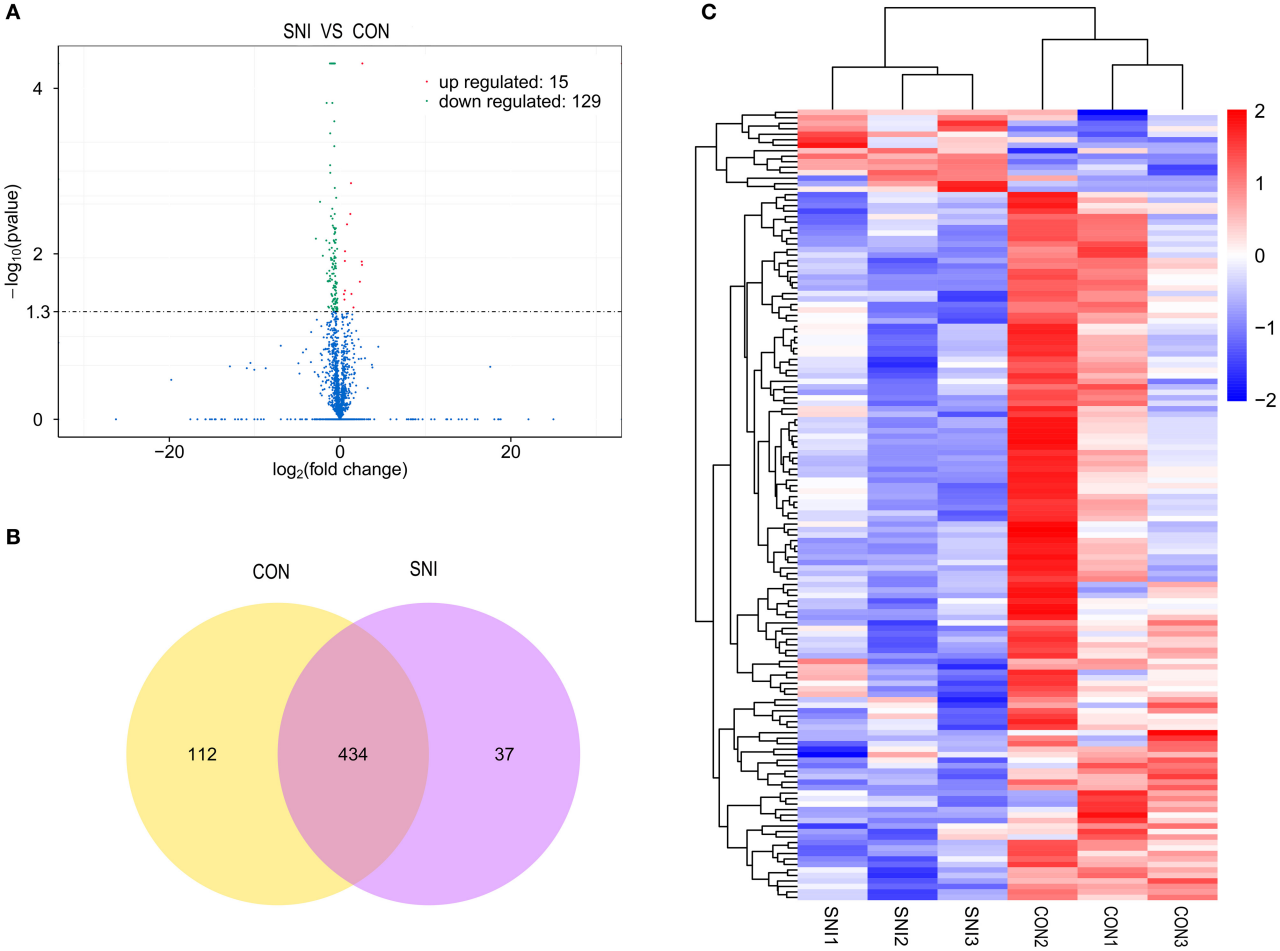
**The expression profiling changes of lncRNAs in spinal cord of SNI rats Vocalno Plot indicate up and down regulated lncRNAs of rats in group SNI compared with group CON (A)**; Venn diagram showing the number of overlap lncRNAs in group SNI compared with group CON **(B)**; Heat map of lncRNAs showing hierarchical clustering of changed lncRNAs of rats in group SNI compared with group CON. In clustering analysis, up- and down-regulated genes are colored in red and blue, respectively **(C)**.

**Figure 3:**
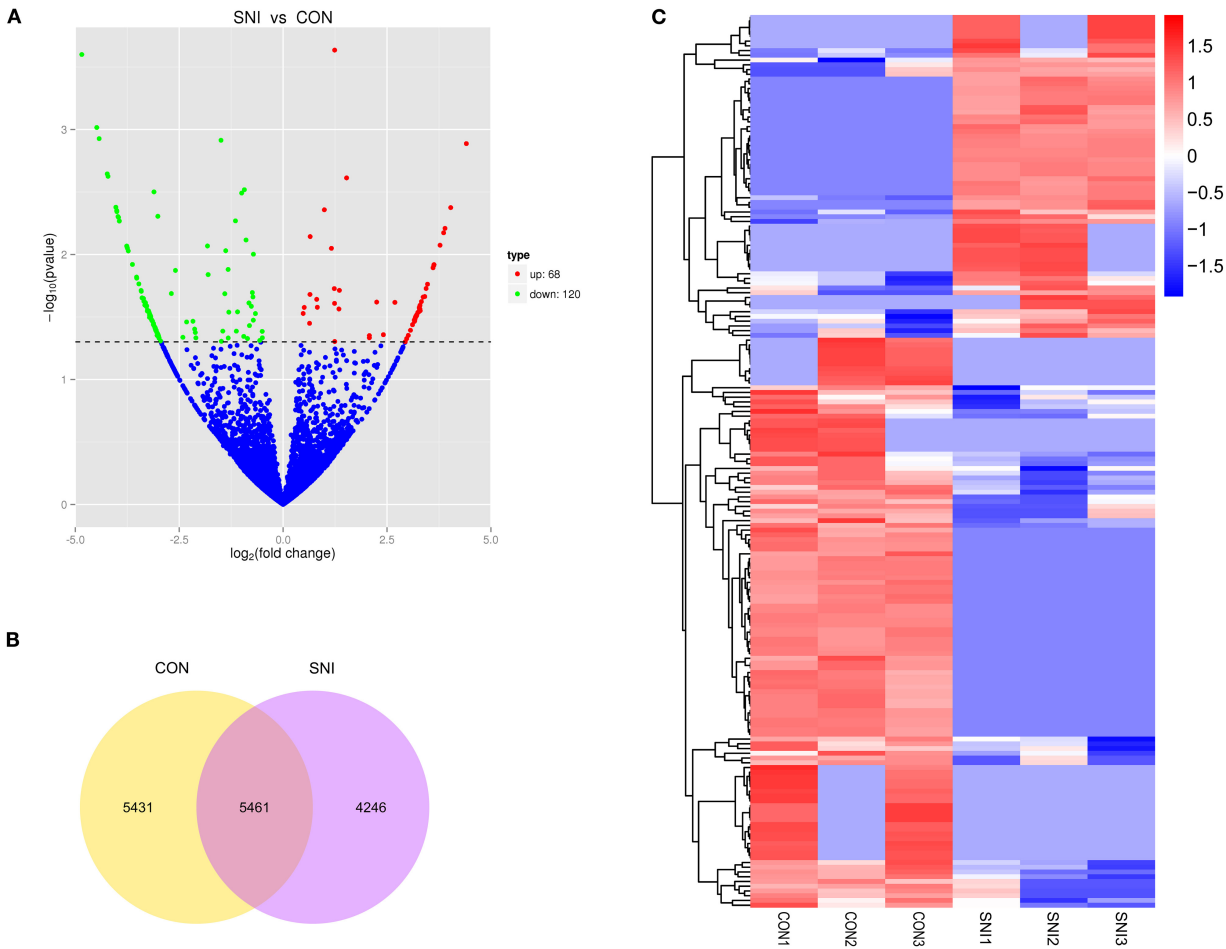
**The expression profiling changes of circRNAs in spinal cord of SNI rats Vocalno Plot indicate up and down regulated circRNAs of rats in group SNI compared with group CON (A)**; Venn diagram showing the number of overlap circRNAs in group SNI compared with group CON **(B)**; Heat map of circRNAs showing hierarchical clustering of changed circRNAs of rats in group SNI compared with group CON. In clustering analysis, up- and down-regulated genes are colored in red and blue, respectively **(C)**.

**Figure 4:**
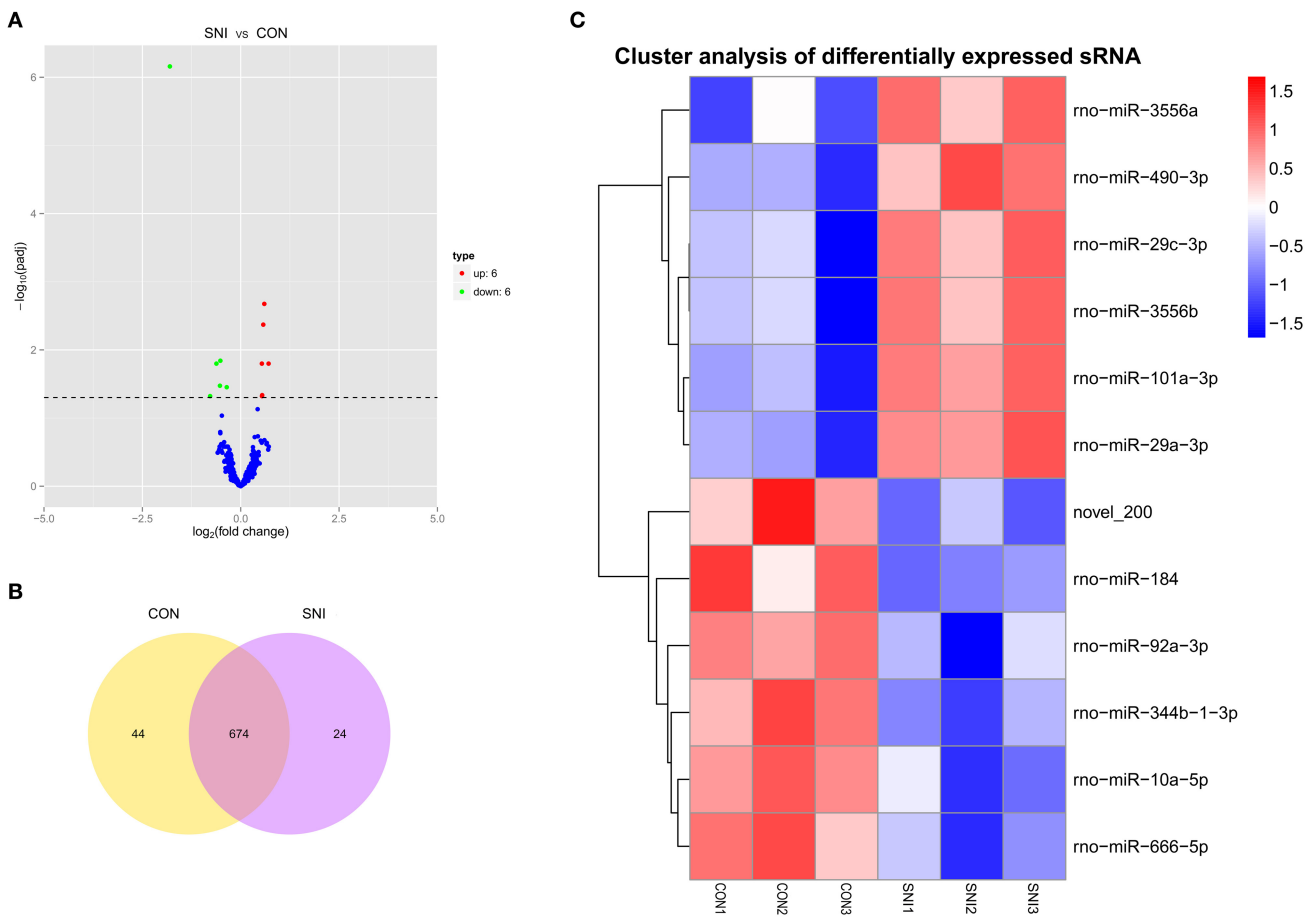
**The expression profiling changes of miRNAs in spinal cord of SNI rats Vocalno Plot indicate up and down regulated miRNAs of rats in group SNI compared with group CON (A)**; Venn diagram showing the number of overlap miRNAs in group SNI compared with group CON **(B)**; Heat map of miRNAs showing hierarchical clustering of changed miRNAs of rats in group SNI compared with group CON. In clustering analysis, up- and down-regulated genes are colored in red and blue, respectively **(C)**.

**Figure 5:**
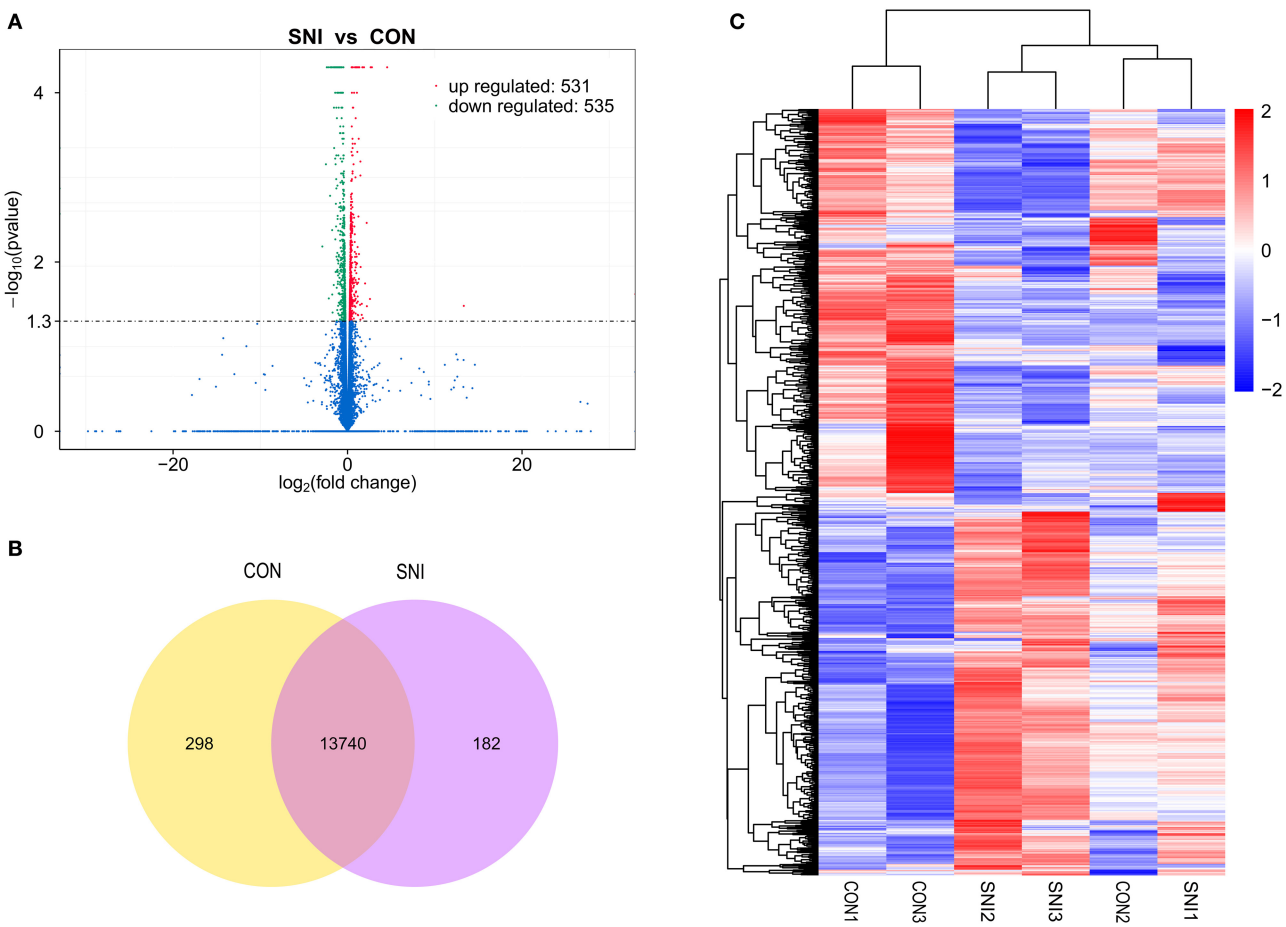
**The expression profiling changes of mRNAs in spinal cord of SNI rats Vocalno Plot indicate up and down regulated mRNAs of rats in group SNI compared with group CON (A)**; Venn diagram showing the number of overlap mRNAs in group SNI compared with group CON **(B)**; Heat map of mRNAs showing hierarchical clustering of changed mRNAs of rats in group SNI compared with group CON. In clustering analysis, up- and down-regulated genes are colored in red and blue, respectively **(C)**.

To determine if ncRNAs are involved in the pathological process of NP, the L_4−5_ spinal cord of rats were analyzed using a sequencing technique at T_4_. We analyzed DE ncRNAs and mRNAs using significance analysis of the microarrays method with Cuffdiff software, following the criteria *q* < 0.05. DE ncRNAs and mRNAs in the samples between T_0_ and T_4_ were shown using a Volcano plot, Venn diagram and clustering map. Information of the top 20 up-regulated and 20 down-regulated lncRNAs, circRNAs, miRNAs, and mRNAs in the SNI group compared with the CON group at 14 days after SNI are listed in Tables 1–4, respectively. All DE miRNAs are listed in Table 3 because only 12 DE miRNAs were detected. Figures 2A–C indicate the Volcano plot, Venn diagram and clustering map of DE lncRNAs, respectively. Figures 3A–C indicate the Volcano plot, Venn diagram and clustering map of DE circRNAs, respectively. Figures 4A–C indicate the Volcano plot, Venn diagram and clustering map of DE miRNAs, respectively. Figures 5A–C indicate the Volcano plot, Venn diagram and clustering map of DE mRNAs, respectively.

**Table 1:** **The detail information of the top 12 up-regulated and 20 down-regulated lncRNAs**.

**Gene id**	**Gene name**	**Gene location**	**SNI FPKM**	**CON FPKM**	**log2 (fold change)**	***p*-value**	**Status**	**Regulation**
XLOC_041439	−	chr7:70468813–70486801	3.2152	0.52746	2.60778	0.00005	Novel_lncRNA	up
XLOC_022312	−	chr19:34624849–34698838	0.650309	0.268292	1.27732	0.0014	Novel_lncRNA	up
XLOC_004676	−	chr1:141625961–141645569	0.656723	0.286778	1.19535	0.0033	Novel_lncRNA	up
ENSRNOG00000051257	LOC100911498	chrX:75109507–75150723	65.6728	37.3181	0.815419	0.0044	Annotated_lncRNA	up
XLOC_021333	−	chr18:60386116–60392321	3.40438	2.3123	0.558064	0.0122	Novel_lncRNA	up
XLOC_043955	−	chr8:46924193–46925663	0.523702	0.0902822	2.53623	0.0124	Novel_lncRNA	up
XLOC_035479	−	chr5:168847439–168849101	0.936985	0.187566	2.32063	0.0217	Novel_lncRNA	up
XLOC_005270	−	chr1:201907283–201966125	34.2383	23.0482	0.570956	0.0278	Novel_lncRNA	up
XLOC_007727	−	chr10:3779415–3781110	0.477579	0.189914	1.33039	0.03055	Novel_lncRNA	up
XLOC_036801	−	chr5:144713816–144730808	7.73259	5.66181	0.449689	0.0307	Novel_lncRNA	up
XLOC_023953	−	chr2:206968570–206974136	4.22487	2.96179	0.512434	0.0357	Novel_lncRNA	up
XLOC_004087	−	chr1:89775068–89788900	7.12858	2.40503	1.56756	0.0445	Novel_lncRNA	up
XLOC_001451	−	chr1:125473010–125489962	1.42892	2.1864	−0.613635	0.00005	Novel_lncRNA	dowm
XLOC_035381	−	chr5:158477328–158487406	0.510472	0.903012	−0.822912	0.00005	Novel_lncRNA	dowm
XLOC_023416	−	chr2:113897921–114011815	0.780907	1.32443	−0.762148	0.00005	Novel_lncRNA	dowm
XLOC_028673	−	chr3:120854547–120878377	2.24016	4.67634	−1.06178	0.00005	Novel_lncRNA	dowm
XLOC_013798	−	chr14:46811773–46814367	1772.1	2971.77	−0.74586	0.00005	Novel_lncRNA	dowm
XLOC_026060	−	chr2:251125838–251137951	0.751836	1.39974	−0.896668	0.00005	Novel_lncRNA	dowm
XLOC_005270	−	chr1:201907283–201966125	17.9187	53.4928	−1.57788	0.00015	Novel_lncRNA	dowm
XLOC_036956	−	chr5:155660093–155664008	1.51401	2.42377	−0.678876	0.0005	Novel_lncRNA	dowm
XLOC_000866	−	chr1:79818164–79873248	0.254892	0.564404	−1.14684	0.00105	Novel_lncRNA	dowm
XLOC_027660	−	chr3:13515049–13534370	0.44141	0.679425	−0.622195	0.0016	Novel_lncRNA	dowm
XLOC_014154	−	chr14:94326327–94329211	0.90401	1.90329	−1.07409	0.0035	Novel_lncRNA	dowm
XLOC_023956	−	chr2:206982504–206984348	1.63981	3.03042	−0.885989	0.00375	Novel_lncRNA	dowm
XLOC_004702	−	chr1:142716628–142721873	0.338685	0.613642	−0.857455	0.00455	Novel_lncRNA	dowm
XLOC_028836	−	chr3:128292384–128311115	0.326965	0.551561	−0.754384	0.0048	Novel_lncRNA	dowm
XLOC_031576	−	chr4:58399806–58415991	0.559063	0.788224	−0.495596	0.0049	Novel_lncRNA	dowm
XLOC_013080	−	chr13:79441798–79484889	0.313276	0.471198	−0.588897	0.00545	Novel_lncRNA	dowm
XLOC_019002	−	chr17:52817364–52846711	0.301231	0.448975	−0.575764	0.00555	Novel_lncRNA	dowm
XLOC_027998	−	chr3:60352440–60353225	0.243615	1.74138	−2.83755	0.00655	Novel_lncRNA	dowm
XLOC_023567	−	chr2:138484146–138490945	1.14949	1.61498	−0.490524	0.00675	Novel_lncRNA	dowm
XLOC_044006	−	chr8:51311534–51318705	0.344242	0.562144	−0.707515	0.007	Novel_lncRNA	dowm

**Table 2:** **The detail information of the top 20 up-regulated and 20 down-regulated circRNAs**.

**ID**	**SNI_readcount**	**CON_readcount**	**log2_FoldChange**	***p*-value**	**Regulation**
rno_circ_0012259	40.53134273	17.27490713	1.2403	0.00023167	up
rno_circ_0006064	6.691544309	0	4.4089	0.0012964	up
rno_circ_0013079	20.65159687	7.221706895	1.527	0.0024384	up
rno_circ_0010349	5.29053236	0	4.0326	0.0042145	up
rno_circ_0006939	76.22276168	38.31478737	0.99228	0.004389	up
rno_circ_0000825	4.906516519	0	3.8952	0.0061775	up
rno_circ_0004918	4.715948174	0	3.8625	0.0067069	up
rno_circ_0011506	91.76729942	58.72960232	0.65096	0.0071894	up
rno_circ_0004059	4.523940254	0	3.7762	0.0084275	up
rno_circ_0005606	21.53969649	9.401199085	1.1619	0.0089261	up
rno_circ_0004527	4.078502251	0	3.6307	0.012054	up
rno_circ_0007564	4.078898877	0	3.6259	0.012204	up
rno_circ_0000377	4.01484726	0	3.6066	0.012775	up
rno_circ_0006860	3.696322611	0	3.4753	0.01728	up
rno_circ_0010435	3.632667621	0	3.4403	0.018708	up
rno_circ_0001732	27.59448536	11.50729162	1.232	0.018738	up
rno_circ_0013883	16.18327631	6.255703379	1.3541	0.019356	up
rno_circ_0000843	62.52054625	40.6386649	0.64624	0.020867	up
rno_circ_0004058	5.417695411	0	3.4084	0.021701	up
rno_circ_0010546	3.567969682	0	3.3685	0.021909	up
rno_circ_0000632	0	9.692558908	−4.8434	0.00025122	down
rno_circ_0010153	0	7.422553095	−4.4831	0.0009639	down
rno_circ_0007461	0	7.261288245	−4.4258	0.0011825	down
rno_circ_0005848	8.540374019	24.83755225	−1.495	0.001219	down
rno_circ_0000631	0	6.487603597	−4.2308	0.0022648	down
rno_circ_0006233	0	6.223179635	−4.2127	0.0023682	down
rno_circ_0005854	35.05074141	68.26107154	−0.93165	0.0030315	down
rno_circ_0000630	0.764359278	8.783191404	−3.107	0.0031568	down
rno_circ_0005849	22.81617484	46.54519374	−0.99699	0.0032257	down
rno_circ_0013893	0	5.490546062	−4.0238	0.004185	down
rno_circ_0004816	0	5.416971244	−4.0024	0.0044553	down
rno_circ_0007269	0	5.52453953	−4.0001	0.0045392	down
rno_circ_0012155	0.764359278	8.36162075	−3.0137	0.0049456	down
rno_circ_0001805	0	5.630638092	−3.9729	0.0049759	down
rno_circ_0004955	0	5.330750937	−3.9598	0.0050454	down
rno_circ_0008945	11.08936446	24.82767105	−1.1456	0.0053833	down
rno_circ_0004776	0	5.487606613	−3.9403	0.0054058	down
rno_circ_0009973	19.88366796	37.25415207	−0.89156	0.0076538	down
rno_circ_0000430	3.313746346	12.6923747	−1.8198	0.008548	down
rno_circ_0012831	0	4.749385158	−3.7624	0.0085598	down

**Table 3:** **The detail information of the up-regulated and down-regulated miRNAs**.

**miRNA**	**SNI readcount**	**CON readcount**	**log2 FoldChange**	***p*-value**
rno-miR-184	118.9078511	685.4126952	−1.8007	0.0000000016245
rno-miR-101a-3p	87300.83865	56528.05376	0.5996	0.0000099004
rno-miR-29a-3p	81902.18011	53999.90849	0.57391	0.000029952
rno-miR-344b-1-3p	554.0495991	805.9714238	−0.51645	0.00013517
rno-miR-490-3p	2729.33079	1588.937044	0.70903	0.00021433
rno-miR-3556a	7962.965229	5376.448171	0.53793	0.00022717
rno-miR-666-5p	144.2955394	228.5159611	−0.61757	0.00026049
rno-miR-92a-3p	4787.869817	7059.746652	−0.52807	0.00062949
rno-miR-10a-5p	287269.4878	369717.8042	−0.35463	0.00074321
rno-miR-29c-3p	1120.817379	747.0056781	0.54598	0.0010812
rno-miR-3556b	1109.862499	742.2301761	0.54095	0.0012748
novel_200	67.29142589	128.2755815	−0.77762	0.00134

**Table 4:** **The detail information of the top 20 up-regulated and 20 down-regulated mRNAs**.

**Gene_id**	**Gene_name**	**Gene_location**	**SNI_FPKM**	**CON_FPKM**	**log2 (fold change)**	***p*-value**	**Regulation**
ENSRNOG00000047098	Hbb-b1	chr1:175104275–175111736	174.178	53.2674	1.70924	0.00005	up
ENSRNOG00000002217	Plac8	chr14:10640285–10661811	9.84226	4.30911	1.1916	0.00005	up
ENSRNOG00000000536	Mdga1	chr20:10707849–10766161	4.0529	1.61119	1.33083	0.00005	up
ENSRNOG00000029886	Hba1	chr10:15497885–15498744	2135.55	1158.7	0.882097	0.00005	up
ENSRNOG00000045840	Hist1h4b	chr4:235021875–235022187	91.2775	43.1417	1.08118	0.00005	up
ENSRNOG00000019183	Alox15	chr10:56704494–56713186	7.5404	1.09044	2.78973	0.00005	up
ENSRNOG00000033307	AABR06091093.1	chr17:29679088–29679871	11.4928	4.65358	1.30432	0.00005	up
ENSRNOG00000026941	Tril	chr4:148632280–148634716	50.5371	36.5662	0.466833	0.00005	up
ENSRNOG00000012067	Fam111a	chr1:236163690–236179257	10.7845	0.463784	4.53936	0.00005	up
ENSRNOG00000048955	LOC689064	chr1:175127945–175129363	805.649	217.996	1.88585	0.00005	up
ENSRNOG00000028756	Ubc	chr12:38515327–38520061	44.4298	29.6808	0.581997	0.00005	up
ENSRNOG00000020951	Slc4a1	chr10:90084740–90101181	1.81462	0.560599	1.69463	0.00005	up
ENSRNOG00000007830	Apold1	chr4:233025349–233028240	8.51911	4.73909	0.846094	0.00005	up
ENSRNOG00000031230	LOC100134871	chr1:175098755–175100086	92.3597	36.2871	1.34781	0.00005	up
ENSRNOG00000007871	Cabp7	chr14:85614858–85655064	100.241	70.5742	0.50626	0.00005	up
ENSRNOG00000046834	C3	chr9:8728464–8754412	22.2468	15.2411	0.545634	0.00005	up
ENSRNOG00000045989	LOC287167	chr10:15472254–15473102	44.1364	7.04644	2.647	0.00005	up
ENSRNOG00000003253	Qdpr	chr14:70207852–70221468	552.873	361.491	0.612989	0.00005	up
ENSRNOG00000013793	C1qtnf9	chr15:44886442–44893075	9.31745	4.50228	1.04928	0.00005	up
ENSRNOG00000005392	Ngfr	chr10:83198414–83216629	3.48169	2.06598	0.752963	0.0001	up
ENSRNOG00000004882	Capn6	chrX:113502311–113527023	1.95767	3.62007	−0.886883	0.00005	down
ENSRNOG00000012660	Postn	chr2:163331261–163362444	0.363375	1.38256	−1.92782	0.00005	down
ENSRNOG00000033090	Ltbp1	chr6:31097089–31491806	1.16404	2.2733	−0.965643	0.00005	down
ENSRNOG00000009345	Ugt8	chr2:248998870–249072741	252.171	409.854	−0.700711	0.00005	down
ENSRNOG00000012404	Thrsp	chr1:168587725–168592099	5.91297	22.787	−1.94626	0.00005	down
ENSRNOG00000019648	Col6a3	chr9:97620135–97670009	0.595406	1.33337	−1.16313	0.00005	down
ENSRNOG00000008749	Col5a1	chr3:11789102–11935751	1.25084	2.11874	−0.760313	0.00005	down
ENSRNOG00000002382	LOC100911714	chr10:47538039–47540977	1.41185	3.43049	−1.28082	0.00005	down
ENSRNOG00000003357	Col3a1	chr9:51689491–51725414	9.31702	15.9013	−0.771204	0.00005	down
ENSRNOG00000015902	Cpxm2	chr1:211048999–211083066	0.887503	2.15548	−1.28019	0.00005	down
ENSRNOG00000012258	Rras2	chr1:185908263–185978450	7.19718	11.6749	−0.697911	0.00005	down
ENSRNOG00000020369	Igf2	chr1:222722921–222730430	12.3147	30.8985	−1.32716	0.00005	down
ENSRNOG00000019050	Ifit1	chr1:260165587–260166976	0.999459	3.42301	−1.77605	0.00005	down
ENSRNOG00000029911	Cilp	chr8:70452357–70467298	0.219025	0.756807	−1.78883	0.00005	down
ENSRNOG00000001267	Lss	chr20:15000295–15027560	12.7964	23.6096	−0.883631	0.00005	down
ENSRNOG00000002916	Car4	chr10:72179404–72188138	2.56717	5.72995	−1.15834	0.00005	down
ENSRNOG00000013717	Bmp6	chr17:28869727–29029564	3.31953	6.22583	−0.907288	0.00005	down
ENSRNOG00000000451	RT1-Ba	chr20:6146270–6150864	4.08884	7.86158	−0.943129	0.00005	down
ENSRNOG00000003172	Serpinf1	chr10:61954920–61969094	1.25674	3.54586	−1.49645	0.00005	down
ENSRNOG00000002918	FAM187A	chr10:90761119–90762587	0.98584	2.61414	−1.40691	0.00005	down

The results of the DE ncRNAs are shown as follows: there were 144 DE lncRNAs (15 up-regulation and 129 down-regulation), 188 DE circRNAs (68 up-regulation and 120 down-regulation) and 12 DE miRNAs (6 up-regulation and 6 down-regulation) in the rat spinal cord at 14 days after SNI in the SNI group compared to the CON group, respectively. The results of the DE mRNAs are shown as follows: There were 531 up-regulated mRNAs and 535 down-regulated mRNAs at 14 days after SNI in the SNI group compared to the CON group, respectively.

**Figure 6:**
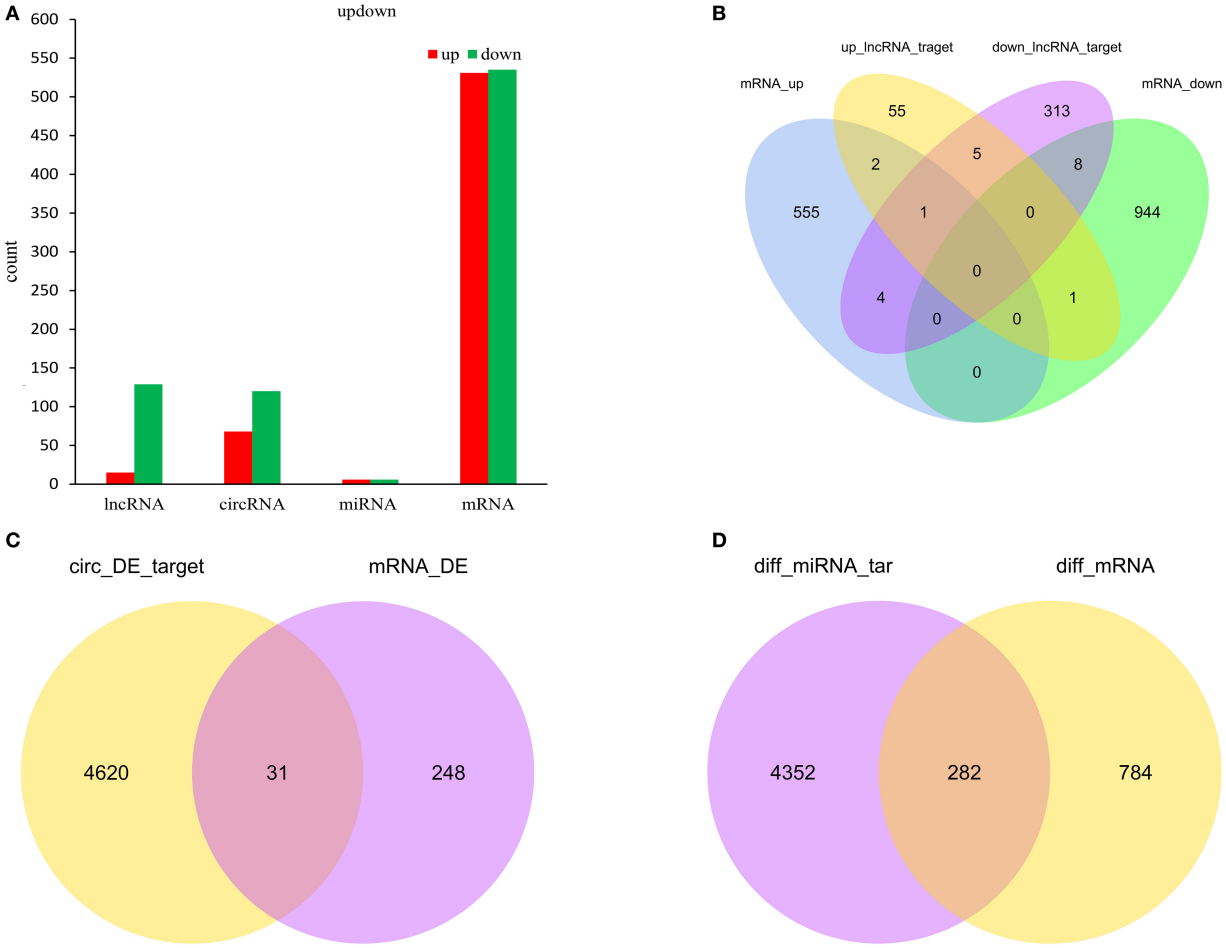
**Count of relatived ncRNAs and mRNAs in spinal cord of SNI rats**. Histogram showing the number of up and down regulated ncRNAs and mRNAs in spinal cord of SNI rats **(A)**; Venn diagram showing the overlap number of targeted mRNA of up-regulated lncRNAs, targeted mRNA of down-regulated lncRNAs, up-regulated mRNAs and down-regulated mRNAs **(B)**; Venn diagram showing the overlap number of targeted mRNA of DE circRNAs and DE mRNAs **(C)**; Venn diagram showing the overlap number of targeted mRNA of DE miRNAs and DE mRNAs **(D)**.

Figure 6A showed the summary histogram of DE lncRNA, DE circRNA, DE miRNA, and DE mRNA.LncRNAs regulated the expression of target gene (mRNA) expression by co-localization or co-regulation. When target genes of lncRNAs are the same as the DE mRNAs, the DE mRNAs could be more directly or indirectly controlled by lncRNAs. Intersectional analysis between target mRNAs of the co-localization or co-expression with lncRNAs and DE mRNAs are shown in the Venn diagram (Figure 6B). Take the intersection of DE mRNAs with DE circRNAs (Figure 6C) and DE miRNAs (Figure 6D) to obtain the information that whether DE circRNAs and DE miRNAs could reflect the change of corresponding DE mRNAs.

### Real-time quantitative polymerase chain reaction (qPCR) validation of ncRNAs and mRNAs expression

**Figure 7:**
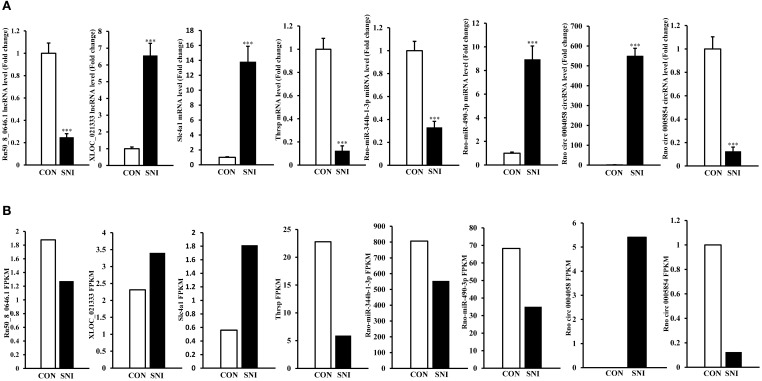
**QPCR validations of eight regulated ncRNAs in the spinal cord from SNI rats**. The expressions of lncRNAs, circRNAs, miRNAs and mRNAs **(A)** were significantly regulated at 14 days after SNI. One-way ANOVA followed by Tukey's multiple comparison test. ^***^P < 0.001. The sequencing results of corresponding lncRNAs, circRNAs, miRNAs and mRNAs with qPCR verification were showed in **(B)**.

To validate the reliability of the sequencing results and to provide the research basis for further study, the changes of some ncRNAs and mRNAs expression at 14 days after SNI were analyzed. The lncRNA (XLOC_021333 and Rn50_8_0646.1), the circRNAs (rno circ 0004058 and rno circ 0005854), the miRNAs (rno-miR-344b-1-3p and rno-miR-490-3p) and the mRNAs (Slc4a1 and Thrsp) were analyzed by qPCR (Figure 7A). The expression levels of corresponding ncRNAs and mRNAs by sequencing method were showed in Figure 7B. All of the validated ncRNAs and mRNAs were consistent with the results obtained from second generation sequencing.

### Functional prediction of DE ncRNAs in SNI

To examine the gene function of ncRNAs in NP, we further selected genes in which the absolute value of correlation was >0.95 to predict the function of ncRNAs using GO and KEGG pathway analysis. LncRNA might be play a regulative role with the nearby protein-coding genes. We set the threshold of co-localization as 100 kb with upstream and downstream of lncRNAs, and then major function of DE lncRNAs were predicted with enrichment analysis.

**Figure 8:**
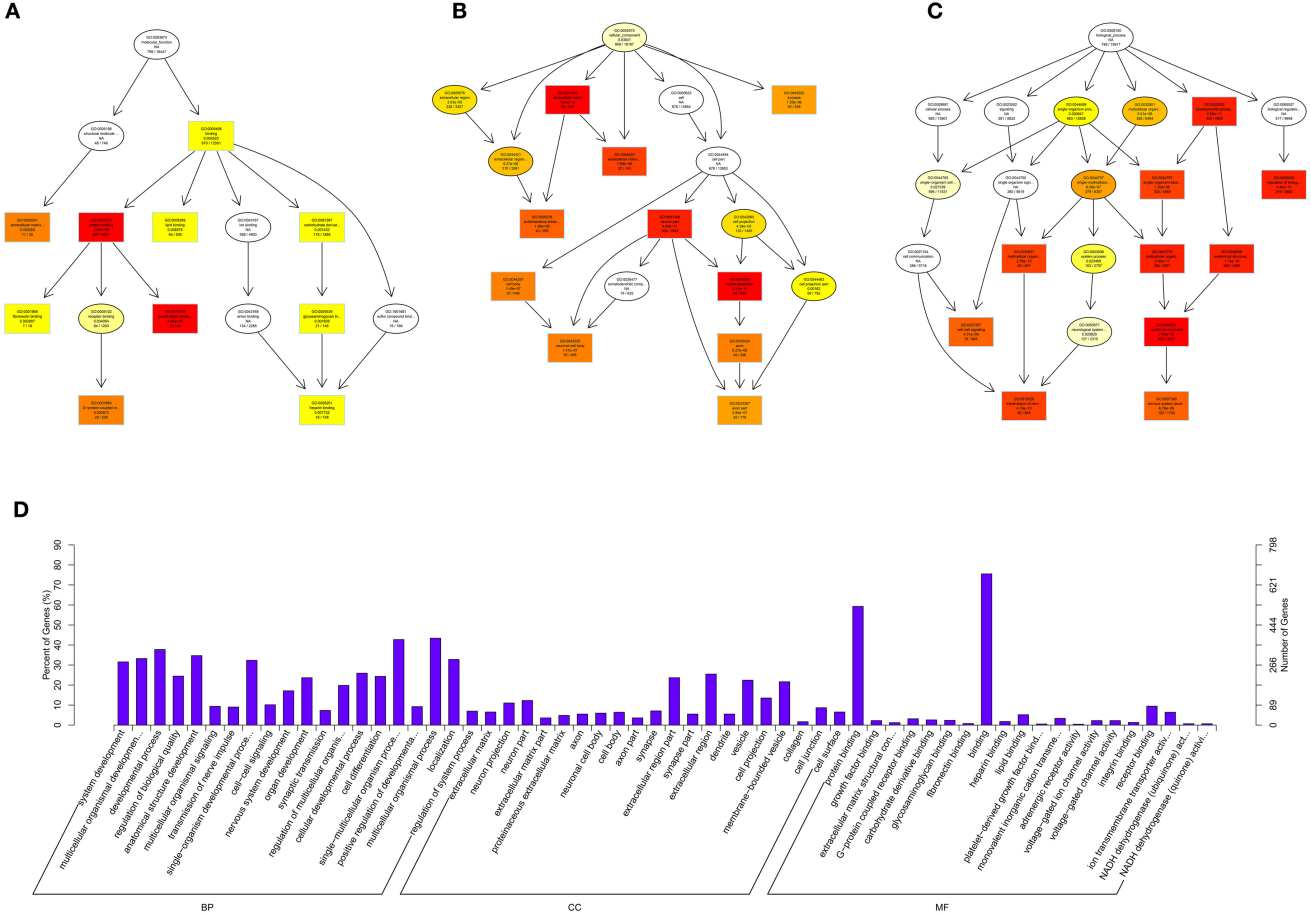
**GO analysis of lncRNAs in spinal cord of SNI rats**. The significant molecular function, biological process and cellular component changed mRNAs targeted lncRNAs in spinal cord of SNI rats. Directed Acyclic Graph (DAG) is the graphical display of GO enrichment results with candidate targeted genes **(A–C)**. The number of genes in GO term were showed in histograph **(D)**.

**Figure 9:**
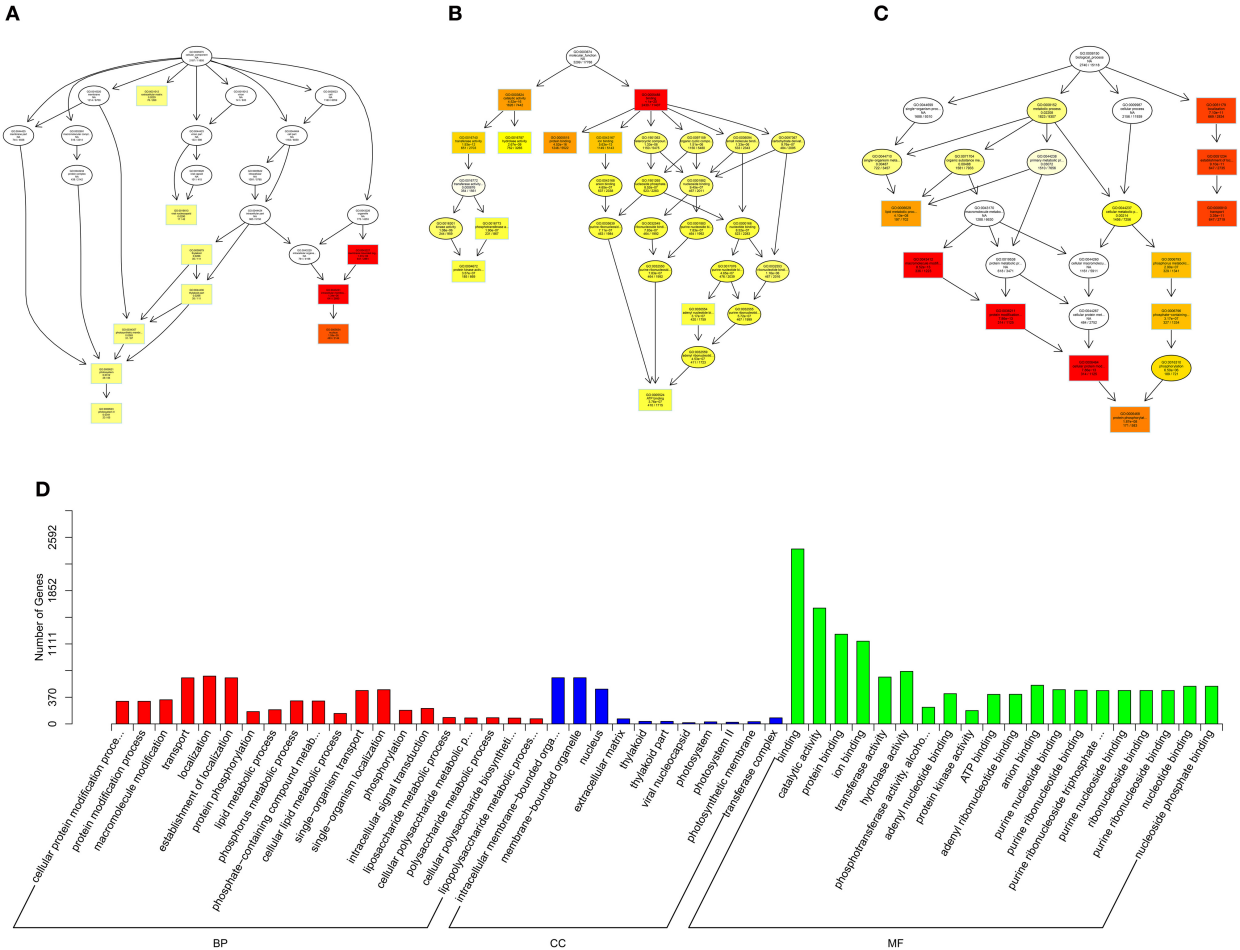
**GO analysis of miRNAs in spinal cord of SNI rats**. The significant molecular function, biological process and cellular component changed mRNAs targeted miRNAs in spinal cord of SNI rats. Directed Acyclic Graph (DAG) is the graphical display of GO enrichment results with candidate targeted genes **(A–C)**. The number of genes in GO term were showed in histograph **(D)**.

**Figure 10:**
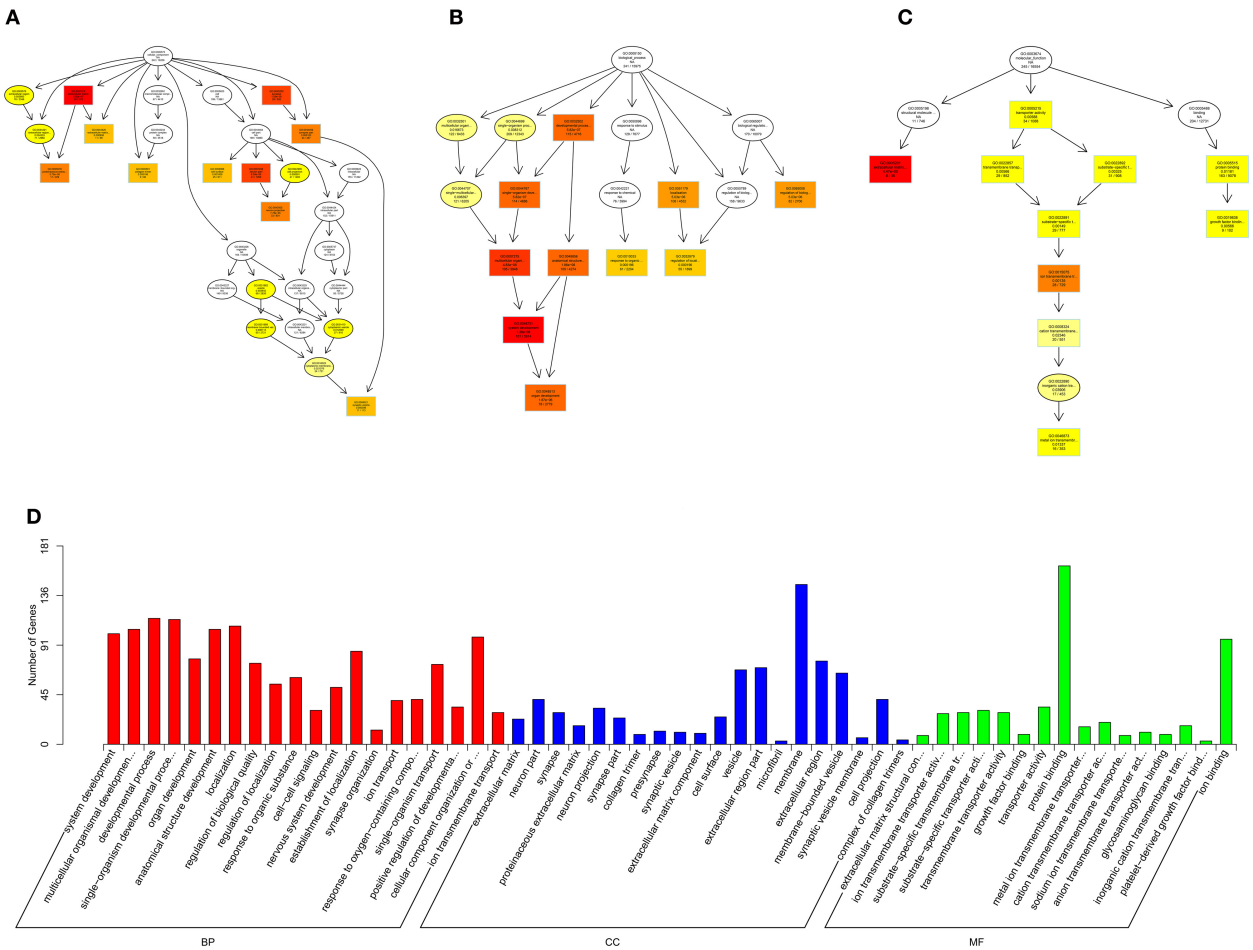
**GO analysis of lncRNA-micRNA-mRNA in spinal cord of SNI rats**. The significant molecular function, biological process and cellular component with changed lncRNA-micRNA-mRNAs in spinal cord of SNI rats. Directed Acyclic Graph (DAG) is the graphical display of GO enrichment results with candidate targeted genes **(A–C)**. The number of genes in GO term were showed in histograph **(D)**.

**Figure 11:**
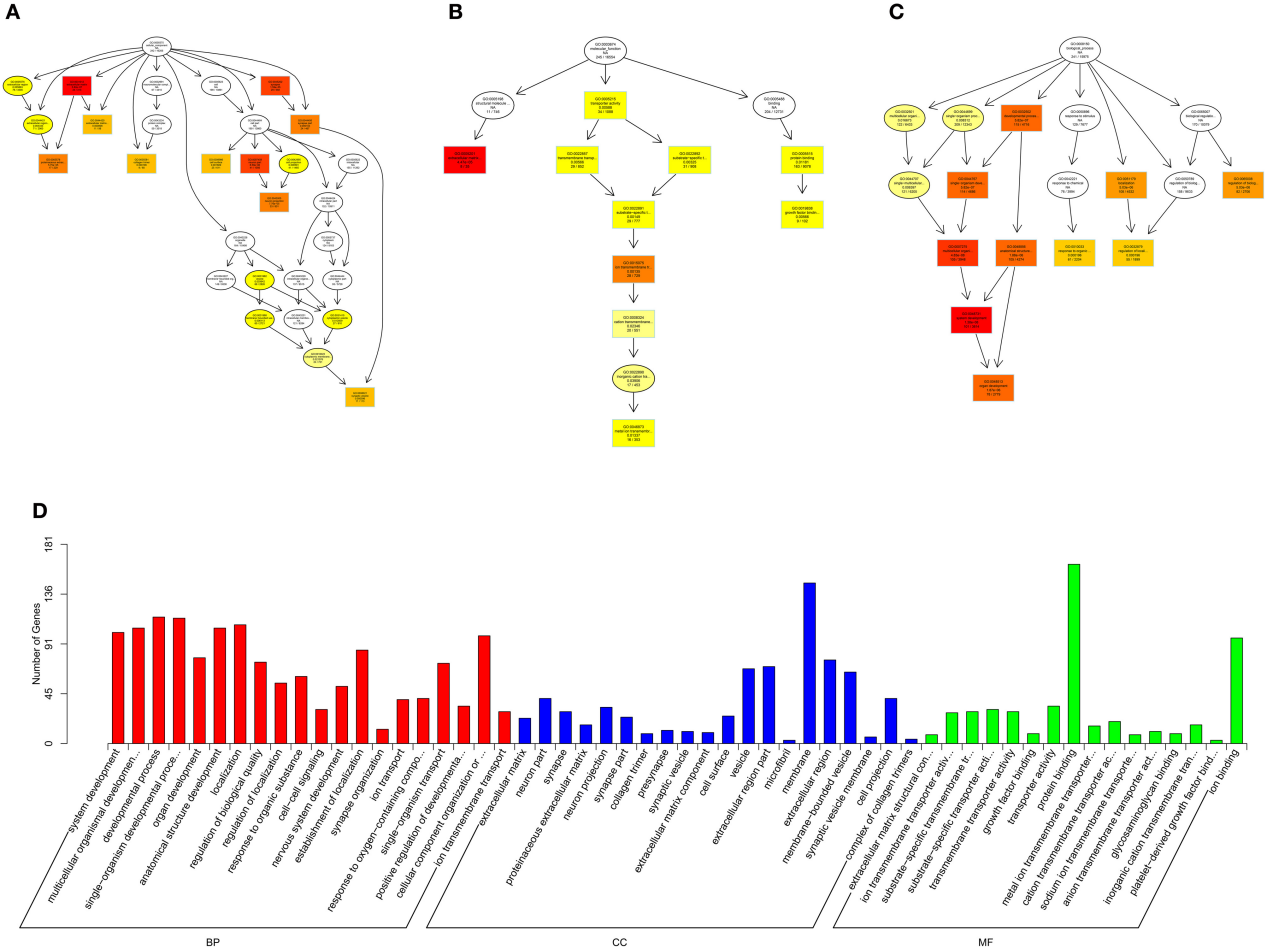
**GO analysis of circRNA-micRNA-mRNA in spinal cord of SNI rats**. The significant molecular function, biological process and cellular component with changed circRNA-micRNA-mRNAs in spinal cord of SNI rats. Directed Acyclic Graph (DAG) is the graphical display of GO enrichment results with candidate targeted genes **(A–C)**. The number of genes in GO term were showed in histograph **(D)**.

Gene Ontology (GO, http://www.geneontology.org/) is the international standard classification system of gene function (Young et al., 2010). Directed acyclic graph (DAG) is a graphical display of DE genes GO enrichment analysis results. The branch represents the relationship of inclusion, which defines the scope from increasingly small from top to bottom. The top 10 results of GO enrichment analysis are selected as the master node of DAG, and are shown together related by GO term by including the relationship, and systematically GO term shown together, where the color depth represents the enrichment degree. DAGs were drawn in the biological process (BP), cellular component (CC) and molecular function (MF), respectively. The DAG of BP, CC and MF, which GO enrichment analysis by co-localization and co-expression of genes of DE lncRNAs are shown in Figures 8A–C. The DAG of BP, CC and MF, which GO enrichment analysis by target genes of DE miRNAs are shown in Figures 9A–C. The DAG of BP, CC and MF, in which GO enrichment analysis by intersection of co-localization and co-expression of genes of DE lncRNAs, target genes of DE miRNAs and predicted mRNAs are shown in Figures 10A–C. The DAG of BP, CC and MF, in which GO enrichment analysis by intersection of targeted genes of DE circRNAs, target genes of DE miRNAs and predicted mRNAs are shown in Figures 11A–C.

According to the distribution of predicted target genes in the Gene Ontology, the gene function of DE ncRNAs were clarified. The number of genes was statistically analyzed with significant enrichment of each GO term, and displayed in the form of a histogram. On the basis of the GO analysis of the co-localization and co-expression genes of DE lncRNAs, the most significantly enriched BP were developmental process, localization and multicellular organismal development, and the most noteworthy enriched CC were membrane-bounded vesicle, extracellular region and extracellular region part. The most significantly enriched MF were binding, protein binding and receptor binding (Figure 8D). Based on the GO analysis of targeted genes of DE miRNAs, the most significantly enriched BP were localization, transport and establishment localization, and the most noteworthy enriched CC were intracellular membrane-bounded organelle, membrane-bounded organelle and nucleus. The most significantly enriched MF were binding, catalytic activity and protein binding (Figure 9D). Based on the GO analysis of intersection of co-localization and co-expression of genes of DE lncRNAs, target genes of DE miRNAs and predicted mRNAs, the most significantly enriched BP were developmental process, single-organism developmental process and localization, and the most noteworthy enriched CC were membrane, extracellular region and extracellular region part. The most significantly enriched MF were protein binding, ion binding and transporter activity (Figure 10D). Based on the GO analysis of intersection of targeted genes of DE circRNAs, target genes of DE miRNAs and predicted mRNAs, the most significantly enriched were the same as the analysis results obtained from co-localization and co-expression of genes of DE lncRNAs, target genes of DE miRNAs and predicted mRNAs (Figure 11D). The most striking category of gene function will be the focus in a future study.

**Figure 12:**
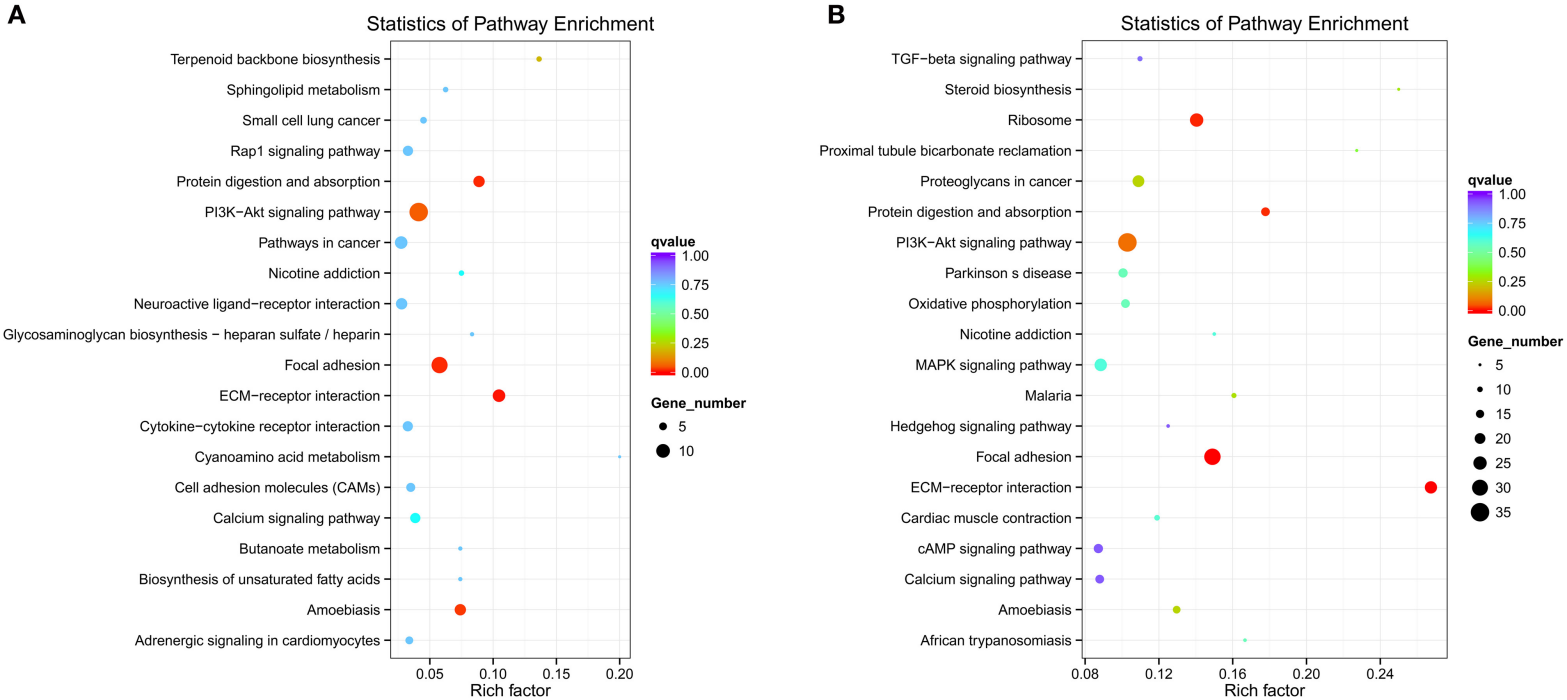
**ncRNAs and mRNAs enriched KEGG pathway scatterplot in spinal cord of SNI rats**. A,B,C,D co-location; E,F,G,H co-expression. LncRNA-micRNA-mRNAs enriched KEGG pathway scatterplot showing statistics of pathway enrichment in spinal cord of SNI rats **(A)**, mRNAs enriched KEGG pathway scatterplot showing statistics of pathway enrichment in spinal cord of SNI rats **(B)**.

Different genes coordinate with each other to exercise their biological functions in the organism. The main biochemical pathways and signal transduction pathways involved with the candidate target genes can be determined through the pathway of significant enrichment. KEGG (Kyoko Encyclopedia of Genes and Genomes) is a major public database on pathways, which can adopt KEGG Pathway as a unit, apply the hypergeometric test, and determine the significant enrichment pathway in the candidate target genes compared with the entire genome background (Mao et al., 2005; Kanehisa et al., 2008). An enriched scatter diagram of the candidate target genes is the graphic display of KEGG enrichment analysis. In this graphic, the degree of KEGG enrichment is assessed by the Rich factor, *Q*-value and number of genes. When the rich factor is greater, the *Q*-value is closer to zero, and the number of genes is greater, then the enrichment is more significant. The top 20 pathways were displayed in the figure. When the data were KEGG-analyzed with intersection of co-localization and co-expression of genes of DE lncRNAs, target genes of DE miRNAs and predicted mRNAs, the most significantly involved pathways in SNI pathogenesis were ribosome, Phosphatidyl Inositol 3-kinase (PI3K)-Akt signaling pathway, focal adhesion, extracellular matrixc (ECM)-receptor interaction, amoebiasis and protein digestion and absorption (Figure 12A). These results were similar with enriched pathways, which were analyzed with predicted genes in SNI pathogenesis (Figure 12B). The main biochemical pathways and signal transduction pathways determined by KEGG analysis will provide further insight toward future research directions of ncRNAs.

### Regulatory network of ncRNA and mRNA

**Figure 13:**
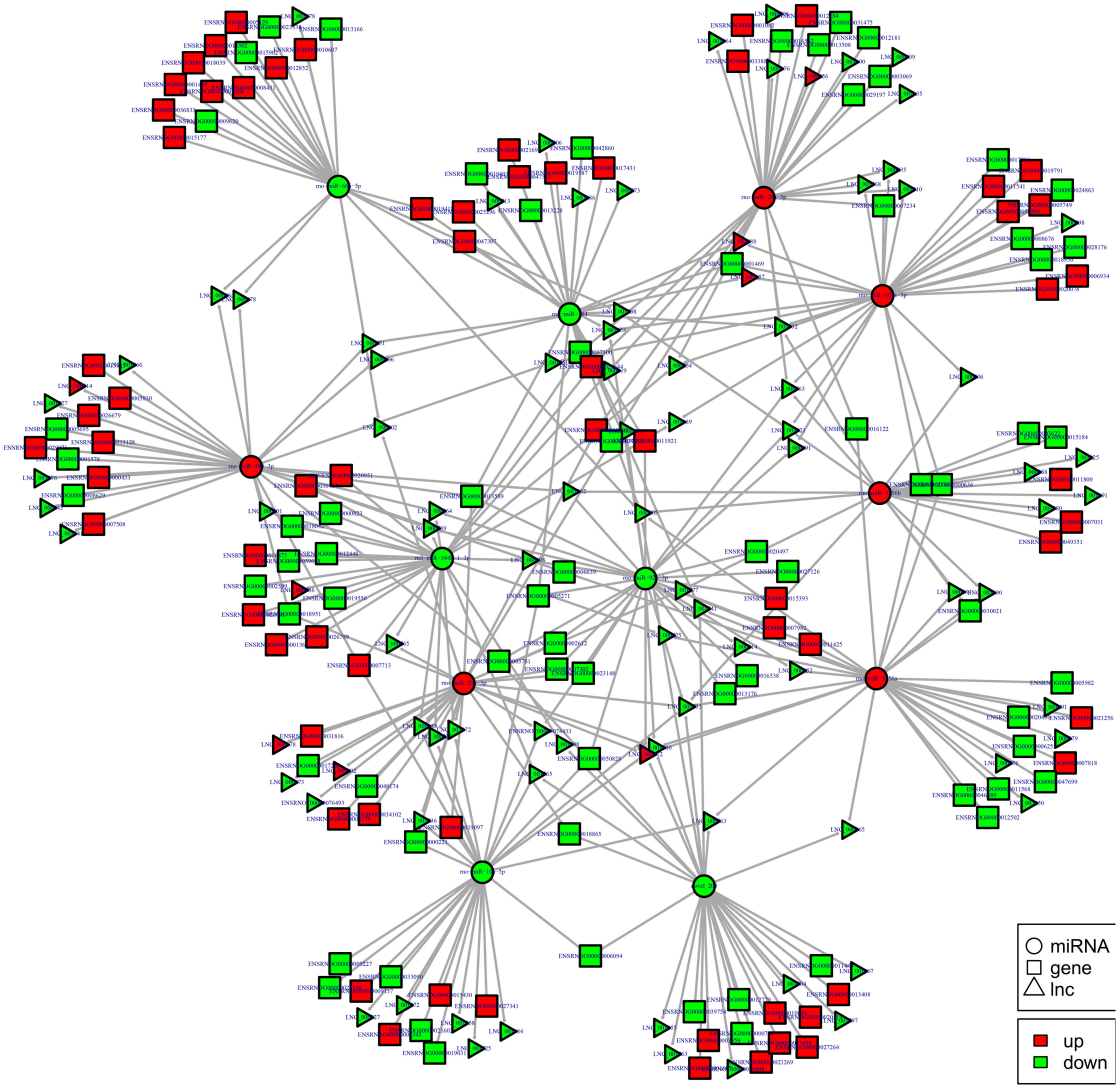
**lncRNA-micRNA-mRNAs regulatory network analysis of ncRNAs in spinal cord of SNI rats**. Figrue 13 is interaction network of lncRNA-micRNA-mRNAs in spinal cord of SNI rats.

**Figure 14:**
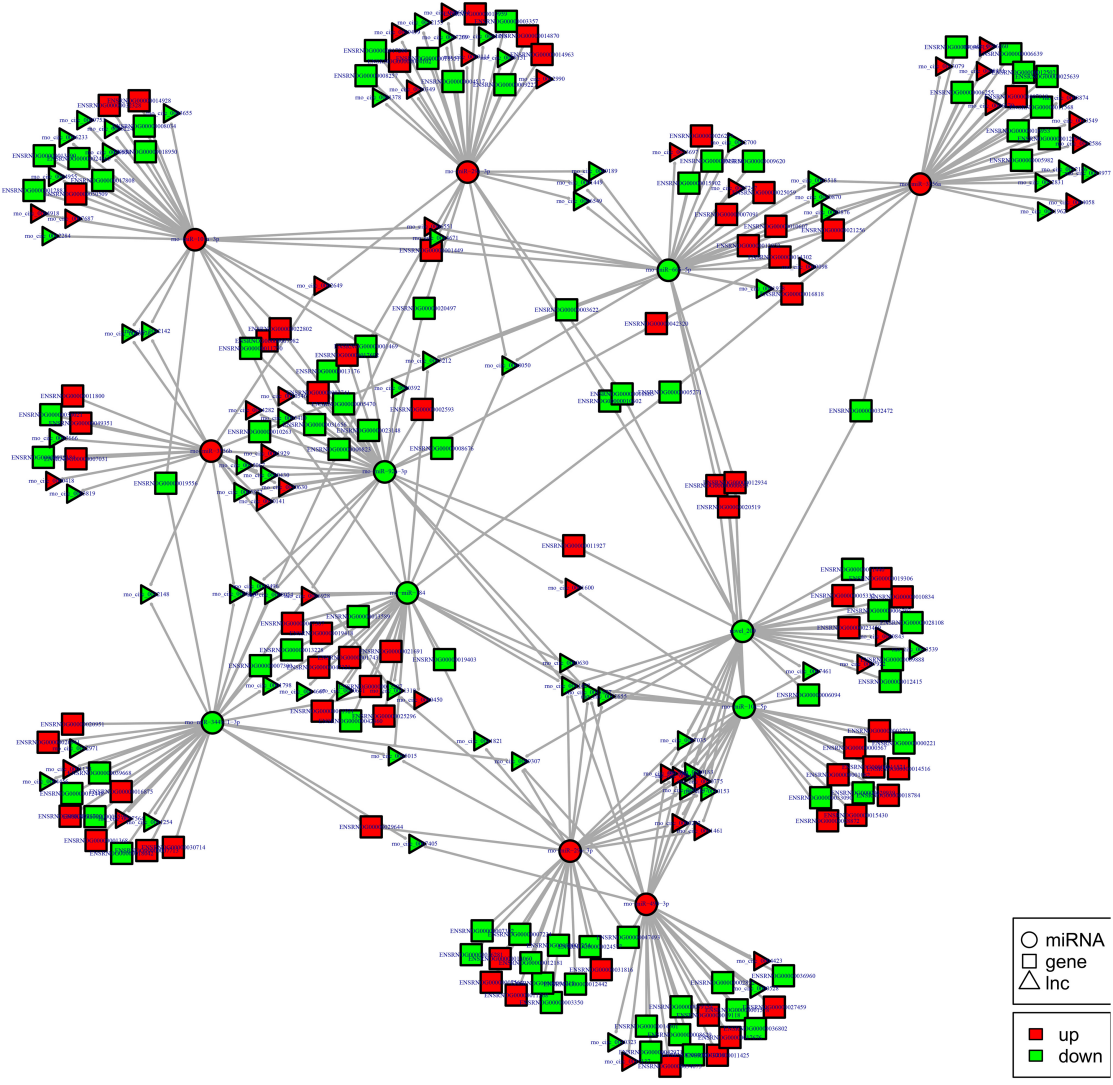
**cirRNA-micRNA-mRNAs regulatory network analysis of ncRNAs in spinal cord of SNI rats**. Figrue 14 is interaction network of circRNA-micRNA-mRNAs in spinal cord of SNI rats.

To examine the molecular mechanism in ncRNAs involved in NP pathogenesis, we performed regulatory network analysis of ncRNA and mRNA in SNI pathogenesis. LncRNA has extensive regulatory function, and it can not only directly regulate the structure of DNA and transcription and translation of RNA, but it can also inhibit target gene regulation of miRNA to indirectly regulate gene expression, thereby acting as a miRNA sponge to bind miRNA competitively with its binding sites. Based on the theory of competing endogenous RNA (ceRNA), lncRNA-gene pairs with same binding sites of miRNA were identified. By constructing lncRNA–miRNA-gene pairs with lncRNA as a decoy, miRNA as the center, mRNA as the target, the ceRNA regulation network were built (Figure 13). Because there are binding sites between the circRNA and miRNA, the regulatory role for the miRNA target gene was inhibited and the gene expression were indirectly regulated by circRNA, which competitively bound the miRNA as a miRNA sponge. Based on the theory of ceRNA, circRNA-gene pairs with same binding sites of miRNA were identified. By constructing circRNA–miRNA-gene pairs with circRNA as a decoy, miRNA as center, mRNA as target, the ceRNA regulation network were generated (Figure 14). The mechanism of gene expression regulated by ncRNA was revealed at the full transcriptome level via the regulatory network of the ceRNA. These results illustrate the regulatory relationship between ncRNA and mRNA in the mechanism of NP. Indeed, as previously described above, the regulatory role of ncRNAs in NP pathogenesis was very complicated such that an in-depth study should be implement in the future.

**Figure 15:**
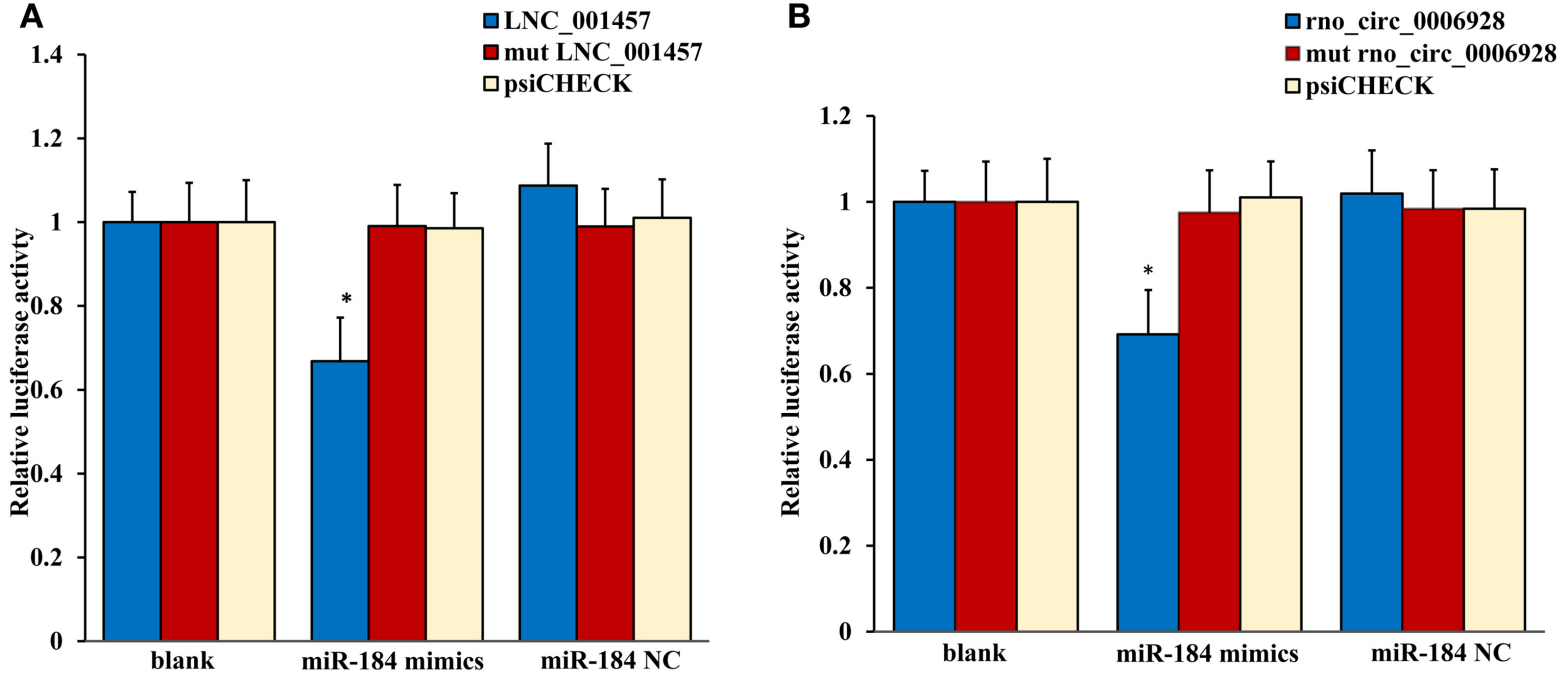
**Confirmation of the pairs relationship**. Luciferase assays using reporter constructs lncRNA (LNC_001457) **(A)** or cirRNA (rno_circ_0006928) **(B)** were performed in NSCs transfected with miRNA-184 (or a control). n = 5, ^*^p < 0.05 compared to the blank group.

The most common mode of action with ncRNA pairs is sponging effect as ceRNA. Considering the apoptosis of neurons in spinal cord play an important role in NP, and miR-184 had been confirmed to be involved in cell apoptosis (Bi et al., 2016). Therefore, we selected two pairs (miR-184 and LNC_001457, miR-184 and rno_circ_0006928) to verify in neural stem cells of rat with dual-luciferase reporter system. Luciferase assay revealed that miR-184 displayed a sponging effect for LNC_001457 (Figure 15A) and crno_circ_0006928 (Figure 15B) and decreased luciferase activity. Acturally, miR-184 was downregulated in SNI in Table 3 and LNC_001457 and circ_0006928 were upregulated in Figures 13, 14, respectively. These results were consistent with our speculation. On the one hand, the results verified the accuracy of the network interaction of ncRNAs. On the other hand, although the sequencing experiment is not a single cell line, the data combined with references of cell function could effectively compensated for the insufficient in the follow-up study.

## Discussion

In the present study, we determined that some ncRNAs and mRNAs were induced significant changes in the spinal cord in response to SNI-induced NP. We also predicted potential functions of DE ncRNAs using GO and KEGG pathway analysis in the SNI model. In addition, the regulatory networks of ncRNA and mRNA were constructed. These findings prompted the proposal that ncRNAs played a significant role in NP processing, and sequencing analysis revealed a potential therapeutic target of NP.

Neuropathic pain (NP) is a debilitating disease that affects the central and peripheral nervous systems, (Zhuo et al., 2011). In addition to spontaneous pain and hyperalgesia, a debilitating symptom of NP is pain hypersensitivity to normally innocuous stimuli, known as tactile allodynia (Baron, 2006). The development of NP is a complex mechanism and is still poorly understood. Future studies of the NP mechanism may have important clinical implications and combined treatments may provide insight toward new therapeutic targets. The spinal dorsal horn receives sensory information from the peripheral nervous system following nociceptive stimuli (Prescott et al., 2014). The pain pathway of the spinal dorsal horn critically contributes to pathologically enhance NP processing (Molander and Grant, 1986; Beggs and Salter, 2006). However, the mechanisms of the spinal dorsal horn are complex and have implications. Non-coding RNA (ncRNA) is a class of genetic, epigenetic and translational regulators, which consists of two major classes according to their size: short ncRNA (<200 nt) and long ncRNA (>200 nt). The transcribed genomic output, which consists of diverse classes of ncRNAs, plays important roles and/or are affected by many biochemical cellular processes, and dysregulation of ncRNA is associated with a variety of diseases, including NP (Guttman and Rinn, 2012; Li et al., 2012; Yu et al., 2013). However, how ncRNAs play such an important and complicated role in NP pathogenesis remains unknown. Thus, we investigated DE ncRNAs and predicted the function and regulatory interaction among ncRNAs and mRNAs in SNI-induced NP, which is essential to thoroughly and comprehensively determine how ncRNAs are involved in NP in further studies.

The SNI model was chosen in the present study to examine ncRNAs in the NP process. The SNI model presents consistent and reproducible pain hypersensitivity in the territory of the spared sural nerve by ligating the common peroneal and tibial nerves, and appear to be advantageous in some aspects, such as less tissue damage, consisting of simple and intuitive experiment steps, good behavior reproducibility and more similar clinical manifestations compared to other NP models, such as chronic constrictive injury (CCI), partial sciatic nerve ligation (PSL), and spinal nerve ligation (SNL) (Bourquin et al., 2006). In our study, all SNI model rats developed mechanical allodynia on the ipsilateral side at T_1−4_, and did not show modification of the thermal sensitivity at any time point. We observed a change in hyperalgesia according to the classic behavior characteristics of the SNI model, which was a reliable precondition of the sequencing results. The spinal cord is a crucial site for nociceptive processing, which materializes to receive, process and relay sensory perception and participates in the coordination of sensory-motor output (Guo and Hu, 2014). Numerous cellular and molecular mechanisms, which involve neurons or glia and astrocytes in the spinal cord were proven to play a critical role in the processing of NP (Alfonso Romero-Sandoval and Sweitzer, 2015). Thus, we examined the DE ncRNAs in the spinal cord of rats in the present study, and our results demonstrated that numerous ncRNAs and mRNAs were significantly up- or down-regulated in the spinal cord of rats in response to SNI. These data indicated that the spinal cord, as an important pain signal transmission center, played a critical role in the cellular and molecular mechanisms of ncRNAs in NP.

Findings obtained from a large body of studies have revealed causal roles of microRNAs in chronic pain (Sakai and Suzuki, 2015). Indeed, miRNAs had been frequently reported as a therapeutic potential target of NP in the past few years (Andersen et al., 2014; Heyn et al., 2016). Recent studies have also shown that peripheral noxious stimuli induced changes in the expression of lncRNAs, which are associated with pain hypersensitivity underlying NP (Zhao et al., 2013). Previous research studies performed gene chip studies to screen and predict DE lncRNAs in the spinal cord SNL model mice (Jiang et al., 2015). Although much evidence has been found, no comprehensive analysis on ncRNAs involved in NP has been performed. Thus, we performed second generation sequencing to analyze DE ncRNAs in the spinal cord of rats with SNI induced-NP. Our results showed that a total of 134 lncRNAs, 12 miRNAs, 188 circRNAs, and 1066 mRNAs were significantly dysregulated at 14 days after SNI surgery. These data indicated that many lncRNAs were involved in NP, and these lncRNAs differed from those observed in Bao-Chun Jiang's research study. One reason for this discrepancy is different species used in the study. Another reason is the analytical results from second generation sequencing were more full-scale than the gene chip studies. Because more novel lncRNAs were detected in the present study, this only highlights the lack of previous knowledge regarding the role of lncRNA in NP. Importantly, circRNA are highly stable, circularized long non-coding RNAs. CircRNAs are conserved across species and are specifically enriched in the nervous system (Van Rossum et al., 2016). Numerous circRNAs have been recently identified; however, the biological function of most circRNAs remains unclear. In addition, there are few research studies on circRNAs in NP. Our research study screened 188 DE circRNAs in NP pathogenesis, which provides further insight on the underlying mechanisms of circRNAs in NP.

Although we screened DE ncRNAs in the spinal cord of rats with SNI induced-NP and a few ncRNAs were confirmed in the present study, these underlying mechanisms of ncNRAs in NP are poorly understood. The Gene Ontology (GO) is a major bioinformatics tool that unifies the representation of genes and gene product attributes across all species (Altshuler et al., 2008). GO terms and GO annotations have proven to be good predictors of gene function and trend (Li et al., 2009; Du et al., 2016). KEGG pathway databases store the higher order functional information for systematic analysis of gene functions and are more widely used in current enrichment analysis platforms (Camon et al., 2004). Thus, we analyzed ncRNAs-related gene functions and the corresponding pathways in the spinal cords of SNI induced-NP rats with GO and KEGG term enrichment analyses in the present study. Our data showed that the most significantly involved pathways in SNI pathogenesis were ribosome, PI3K-Akt signaling pathway, focal adhesion, ECM-receptor interaction, amoebiasis and protein digestion and absorption. The function of ncRNAs predicted with GO and KEGG analysis in the mechanisms of NP should be studied more in-depth in future work.

RNA transcripts with microRNA response elements (MREs) might act as ceRNA, which include pseudogene transcripts, lncRNAs, circRNAs and mRNAs, which act as natural miRNA sponges to suppress miRNA function using shared MREs for mutual regulation (Ashwal-Fluss et al., 2014). Although theoretical and experimental studies have partially revealed that lncRNAs and/or miRNAs can regulate gene expression involved in mechanisms of NP (Bali and Kuner, 2014; Xiong et al., 2015), circRNAs appears to be more correlated with pain because they were more enriched in neurons and have been proven to regulate synaptic function (You et al., 2015). However, the molecular mechanisms underlying the interaction of ncRNAs and mRNAs in NP remain largely unclear. Thus, for the first time, the lncRNA-miRNA-mRNA and circRNA-miRNA-mRNA network of NP was constructed based on our second sequencing data. These pioneering discoveries might enrich our understanding on the mechanisms underlying the role of non-coding RNAs in NP pathogenesis.

It is worth mentioning that the spinal cord contains a variety of cell populations, including many kinds of neuron subtypes, astrocytes and microglia. The GO, KEGG and ncRNA regulative network analyses we proposed in this paper just made the spinal cord as a whole. Therefore, lots of experiment *in vitro* and *in vivo* need to further verified and analyzed. The single-celled sequencing analysis with neurons of spinal cord are implementing with our team. Related results will be combined with the current data, can be more accurately described ncRNAs function of neurons or gliocytes in spinal cord in mechanism of NP.

Because lncRNA, miRNA, and circRNAs and mRNA are studied together for their predicted role and regulated relationship, our study presented an innovative data integration analysis of ncRNA and mRNA in NP pathogenesis. We also provided a catalog of predicted ncRNAs in NP and identified and confirmed several genes using sequencing, which may be of relevance for other research groups. These abundant data prompted the proposal that ncRNAs may interact and regulate their related protein-gene expression and play a key role in the pathogenesis of NP. The next step is to further study these predicted ncRNAs with regard to their complete proteomic and relevant signaling pathway, which may ultimately enable the full disclosure of the mechanisms underlying NP.

## Methods

### Animals

Adult male Sprague Dawley rats (body weight 250–280 g, the Laboratory Animal Center of The First People's Hospital of Foshan, Foshan, China) were randomly assigned to standard cages with 4 to 5 animals per cage. All animal procedures were in accordance with national and international animal care and ethical guidelines and have been approved by the institutional animal welfare committee. The environment was maintained at a constant temperature (22 ± 0.581°C) and relative humidity (60–70%) with a 12-h light/dark cycle (lights on at 7 a.m.). All animals were fed with a standard laboratory diet and tap water was provided *ad libitum*. The animals were placed in the experimental room 24 h before behavioral testing for acclimatization.

### SNI model

The rats were fixed to an operating table after anesthesia, and the femur at the right mid-thigh level was used as the incision landmark. The 3 peripheral branches (the sural, common peroneal, and tibial nerves) of the sciatic nerve were exposed at mid-thigh level distal to the trifurcation and exposed without stretching of the nerve structures and freed of connective tissue under sodium pentobarbital anesthesia (45 mg/kg, intraperitoneally). The tibial and common peroneal nerves were tightly ligated by two knots, 4 mm apart, using 6.0 silk (Ethicon, Johnson & Johnson Inc., Brussels, Belgium) and were completely severed between the knots. The sural nerve was left intact. The sural nerve was carefully preserved by avoiding any nerve stretch or contact with surgical tools. The muscle and skin were closed in 2 distinct layers using silk 5.0 suture (Decosterd and Woolf, 2008).

### Nociceptive behavior

All behavioral tests were executed in a blind manner. Nociceptive responses to mechanical and thermal stimuli were measured 3 h before and 1, 3, 7, and 14 days after surgery. Mechanical withdrawal threshold (MWT) was measured to an applied von Frey hair (Stoelting, Wood Dale, Illinois, USA) to the right hind paw until a positive sign of pain behavior was elicited (Chaplan et al., 1994). The test area was the mid-plantar claw in the distribution area of the sciatic nerve. Von Frey filaments with logarithmically incremental stiffness (0.4–15.1 g) were serially applied to the paw using the up-down method. The hairs were presented in ascending order of strength, perpendicular to the plantar surface, with sufficient force to cause slight bending against the paw and were held for 6–8 s. A positive response was noted if the paw was sharply withdrawn. Flinching immediately upon removal of the hair was also considered a positive response. The 15.1-g hair was selected as the upper cutoff for testing. Animals that exhibited no response to the 15.1-g filament were assigned this cutoff value. The bending force that evoked 50% of paw withdrawal occurrence was set as the MWT. The Hargreaves test was used to measure paw thermal withdrawal latency (TWL) to heat stimuli and to determine the presence of thermal hyperalgesia (Hargreaves et al., 1988). The rat was placed on the surface of a 2-mm-thick glass plate covered with a plastic chamber (20 × 20 × 25 cm). TWLs were measured using a radiant thermal stimulator (BME410A, Institute of Biological Medicine, Academy of Medical Science, Tianjin, and China). Heat was concentrated on the hind paw, which was flush against the glass, and radiant heat stimulation was delivered to the site. The stimulus terminated with hind paw movement, and a 25-s cutoff was imposed on the stimulus duration to prevent tissue damage. The intensity of thermal stimulation remained constant throughout the experiment. Five stimuli were applied to the same site, and the average TWL from three thermal stimulations was obtained. This mean TWL was used as the steady state of the TWL value.

### Tissue collection

We prepared 12 rats for SNI and sham operation. At 14 days after operation, three rats were randomly selected in each group and deeply anesthetized with isoflurane after the behavioral test. The L_4−5_ spinal cord segments were collected after perfusion. Total RNA was extracted from the spinal cord dorsal horn tissue using Trizol reagent (Invitrogen, Carlsbad) according to the manufacturer's instructions.

### RNA isolation and RNA quantification and qualification

RNA degradation and contamination was monitored on 1% agarose gels. RNA purity was confirmed using the NanoPhotometer® spectrophotometer (IMPLEN, CA, USA). The RNA concentration was measured using the Qubit® RNA Assay Kit in Qubit® 2.0 Fluorometer (Life Technologies, CA, USA). RNA integrity was assessed using the RNA Nano 6000 Assay Kit of the Bioanalyzer 2100 system (Agilent Technologies, CA, USA). The quantitative method of lncRNAs and miRNAs were same as conventional procession of mRNAs. Quantification of circRNAs should be performed with exonuclease to exclude non-circRNAs. Take two copies of the same amount of RNA extraction, one sample was digested the linear RNA with RNase R (Cat. No. RNR07250, Epicentre Company, USA) and leaved only circRNAs, another sample did not treated with RNase R. The two RNA were processed to reverse transcriptase meanwhile. The samples with RNase treatment were used to detect circRNAs, the samples untreated with RNase were used to detect β-actin. Library preparation for lncRNA sequencing.

A total of 3 μg RNA per sample was used as input material for the RNA sample preparations. First, ribosomal RNA was removed using the Epicenter Ribo-zero™ rRNA Removal Kit (Epicenter, USA), and rRNA-free residue was cleaned up by ethanol precipitation. Subsequently, sequencing libraries were generated using the rRNA-depleted RNA by NEBNext® Ultra™ Directional RNA Library Prep Kit for Illumina® (NEB, USA) following manufacturer's recommendations. Briefly, fragmentation was performed using divalent cations under elevated temperature in NEBNext, First Strand Synthesis Reaction Buffer (5X). First strand cDNA was synthesized using random hexamer primers and M-MuLV Reverse Transcriptase (RNaseH-). Second strand cDNA synthesis was subsequently performed using DNA Polymerase I and RNase H. In the reaction buffer, dNTPs with dTTP were replaced by dUTP. Remaining overhangs were converted into blunt ends via exonuclease/polymerase activities. After adenylation of 3′ ends of DNA fragments, NEBNext, Adaptor with hairpin loop structure were ligated to prepare for hybridization. To select cDNA fragments of preferentially 150–200 bp in length, the library fragments were purified with AMPure XP system (Beckman Coulter, Beverly, USA). Then, 3 μl USER Enzyme (NEB, USA) was used with size-selected, adaptor-ligated cDNA at 37°C for 15 min followed by 5 min at 95°C before PCR. Then, PCR was performed with Phusion High-Fidelity DNA polymerase, Universal PCR primers and Index (X) Primer. Finally, products were purified (AMPure XP system) and library quality was assessed on the Agilent Bioanalyzer 2100 system.

### Library preparation for small RNA sequencing

A total of 3 μg total RNA per sample was used as input material for the small RNA library. Sequencing libraries were generated using NEBNext® Multiplex Small RNA Library Prep Set for Illumina® (NEB, USA.) following the manufacturer's recommendations and index codes were added to attribute sequences to each sample. Briefly, the NEB 3′ SR Adaptor was directly and specifically ligated to the 3′ end of miRNA. After the 3′ ligation reaction, the SR RT Primer hybridized to the excess of 3′ SR Adaptor (that remained free after the 3′ ligation reaction) and transformed the single-stranded DNA adaptor into a double-stranded DNA molecule. This step is important to prevent adaptor-dimer formation. In addition, dsDNAs are not substrates for ligation mediated by T4 RNA Ligase 1 and thus do not ligate to the 5' SR Adaptor in the subsequent ligation step. Next, the 5'end adapter was ligated to 5'ends of miRNAs. First strand cDNA was synthesized using M-MuLV Reverse Transcriptase (RNase H–). PCR amplification was performed using LongAmp Taq 2X Master Mix, SR Primer for Illumina and index (X) primer. PCR products were purified on an 8% polyacrylamide gel (100 V, 80 min). DNA fragments corresponding to 140–160 bp (the length of small noncoding RNA plus the 3′ and 5′ adaptors) were recovered and dissolved in 8-μL elution buffer. At last, library quality was assessed on the Agilent Bioanalyzer 2100 system using DNA High Sensitivity Chips.

### Clustering and sequencing of ncRNA

The clustering of the index-coded samples was performed on a cBot Cluster Generation System using TruSeq PE Cluster Kit v3-cBot-HS (Illumina) according to the manufacturer's instructions. After cluster generation, the libraries were sequenced on an Illumina HiSeq 2500 platform, 125 bp paired-end and 50-bp single-end reads were generated. The reads number of lncRNAs, circRNAs and miRNAs for each samples were, respectively 94210880-113312518, 94210880-113312518 and 14658590-18771808. The clean bases of lncRNAs, circRNAs and miRNAs for each samples were, respectively 13.74G-16.43G, 13.74G-16.43G, and 0.733G-0.939G. The error rate of lncRNAs, circRNAs and miRNAs for each samples were respectively <0.02, <0.02, and <0.01%. The transcription with splicing of each sample were combined and screened as lncRNAs with Cuffmerge Software, and the conditions were as follows: the number of exon≥2, length > 200 bp, FPKM ≥0.5 (Cuffquant) and to eliminate overlapping and coding potential transcription with annotation of database at exon region (Cuffcompare Software). CircRNAs were identified base on the data of lncRNAs with find_circ (Memczak et al., 2013). Clean reads were screened the lengh of 21–22 nt as miRNA, and located to reference sequence with bowtie. Combined with miREvo Software (Wen et al., 2012) and mirdeep2 Software (Friedländer et al., 2012) to analysis the funtions of new miRNAs. Adopt DESeq2 with negative binomial distribution (Love et al., 2014) to analyse differentially expression of ncRNAs All sequencing program were performed by Novogene Company (China, Beijing).

### Quantitative real-time RT-PCR

The remaining three rats in each group were validated using real-time PCR. Spinal cord tissues were separated under anesthetic after the behavioral test. The first-strand cDNA was synthesized using reverse transcription kit (Vazyme) according to the manufacturer's instruction and amplified in triplicate using IQ SYBR green SuperMix reagent (Bio-Rad, Hercules, CA) with an Opticon real-time PCR machine (MJ Research, Waltham, MA), according to the manufacturer's instructions. The specificity of real-time PCR was confirmed via routine agarose gel electrophoresis and melting-curve analysis. The sequences of all primers are shown in Table 5. The comparative Ct method (ΔΔCt) was used to quantify gene expression, and the relative quantification was calculated as 2−ΔΔCt. Calculation of lncRNAs and circRNAs expression were consistent with mRNA, the internal control were β-actin. The expression levels of miRNAs were normalized to U6 level in each sample.

**Table 5:** **Primers designed for qRT-PCR validation of candidate ncRNAs and mRNAs**.

**Gene**	**Primer**	**Product length**
ENSRNOG00000049915	F ACATCAGCAGCCTGCCATTA	160
	R ATCACCTCCCTCTGCTGTGT	
XLOC_021333	F TCGGGATAGCGAAAGTTCT	121
	R CTGATGAGAGACGTTGAGTGA	
Thrsp	F AACATGGAGCAAGTGGTGAT	120
	R GCTCTTGAGCATGGTGAAGTA	
Slc4a1	F TGCCATGATGCTACGAAAGT	150
	R ACCGAGAGTTTCTGCGTGTA	
rno_circ_0005854	F TGCAGAGGAACTGAGTATGG	125
	R CAGGGACTCTGTCCCCTTTC	
rno_circ_0004058	F AAATTATCTCGAATGGAGTG	88
	R GCGAGCATCTCTTCGGACTT	
rno-miR-490-3p	RT CTCAACTGGTGTCGTGGAGTCGGCAATTCAGTTGAGCAGCATGG	72
	F ACACTCCAGCTGGGCAACCTGGAGGACTCCAT	
	R CTCAACTGGTGTCGTGGA	
rno-miR-344b-1-3p	RT CTCAACTGGTGTCGTGGAGTCGGCAATTCAGTTGAGACAGTCGG	72
	F ACACTCCAGCTGGGGATATAACCAAAGCCCGA	
	R CTCAACTGGTGTCGTGGA	
U6	RT AACGCTTCACGAATTTGCGT	94
	F CTCGCTTCGGCAGCACA	
	R AACGCTTCACGAATTTGCG	

### Go annotations and KEGG pathways analysis

Gene Ontology (GO) annotations and KEGG pathway analysis were applied to investigate the roles of all DE ncRNAs. Briefly, GO analysis was applied to elucidate genetic regulatory networks of interest by forming hierarchical categories according to the BP, CC, and MF aspects of the differentially expressed genes (http://www.geneontology.org). Pathway analysis was performed to explore the significant pathways of the differentially expressed genes, according to KEGG (http://www.genome.jp/kegg/).

### Analysis of ncRNAs regulatory network

To reveal the role and interactions among ncRNAs and mRNAs in NP pathogenesis, we constructed a ncRNAs regulatory network. The interaction network was built and visually displayed using Cytoscape software based on the screening of lncRNA-miRNA-gene pairs and circRNA-miRNA-gene pairs with miRanda software and psRobot software. Different shapes represent different RNA types, and different colors represent the regulated relationship. The size of the node was in direct proportion to the degree. Thus, these significant nodes are in a core position in the regulated network, which are more associated with NP.

### Luciferase assay

A dual-luciferase reporter system E1960 (Promega, Madison, WI, USA) was used to perform luciferase activity assay. In brief, embryonic neural stem cells (NSCs) of rats were cultured on 12-well tissue culture plates at a density of 2 × 10^5^ cells per well. Cells were co-transfected with the luciferase reporter constructs contain lncRNA (LNC_001457) or cirRNA (rno_circ_0006928), miRNA(miR-184) mimics and Renilla luciferase construct for 5 h (Lipofectamine® MessengerMAX™ Transfection Reagent, Thermo Fisher Scientific). After 3d culture at 37°C, the transfected cells were lysed by 150 μl of passive lysis buffer. In total, 30 μl of lysates were mixed with 50 μl of LAR II, and then firefly luciferase activity was measured by a luminometer. For the internal control, 50 μl of Stop and Glo reagent was added to the sample.

### Statistical analysis

The data are presented as the means ± SEM. The results from the behavioral study were statistically analyzed using one-way or two-way analysis of variance (ANOVA). The RT-PCR results were analyzed using one-way analysis of variance followed by Tukey's multiple comparison test. Significance was established at *p* < 0.05.

## Ethics statement

The treatment of animals used in the study was approved by the First People's Hospital of Foshan Ethics Committee.

## Author contribution

JZ is responsible for the construction of animal model and collection of results and analysis of ncRNAs regulatory network; QX is responsible for the RNA isolation and RNA quantification and qualification; HC is responsible for the library preparation for lncRNA and Small RNA sequencing and Quantitative Real-Time RT-PCR; CY is responsible for tissues collection and the satistical analysis; YF is responsible for the preparation for lncRNA and Small RNA sequencing and analysis of ncRNAs regulatory network.

## Funding

National Natural Science Foundation of China (81300974), the Natural Science Foundation of Guangdong Province (2015A030313899) and the Medical Scientific Research Foundation of Guangdong Province (A2015013).

### Conflict of interest statement

The authors declare that the research was conducted in the absence of any commercial or financial relationships that could be construed as a potential conflict of interest.
